# Meta-inflammation and endotoxemia in a highly translational porcine model of diet-induced obesity

**DOI:** 10.1038/s41684-025-01588-3

**Published:** 2025-08-06

**Authors:** Sofie M. R. Starbæk, Betina L. Henriksen, Louise Brogaard, Eline E. Jessen, Tim K. Jensen, Steffen Goletz, Peter M. H. Heegaard, Kerstin Skovgaard

**Affiliations:** 1 Department of Biotechnology and Biomedicine, Technical University of Denmark, Kongens Lyngby, Denmark; 2 Department of Veterinary and Animal Sciences, University of Copenhagen, Frederiksberg, Denmark; 3 Department of Health Technology, Technical University of Denmark, Kongens Lyngby, Denmark; 4 Present Address: Mater Research Institute–University of Queensland, Translational Research Institute, Woolloongabba, Queensland Australia

**Keywords:** Obesity, Experimental models of disease, Chronic inflammation

## Abstract

Meta-inflammation (chronic, low-grade systemic inflammation) is increasingly recognized as an essential link between obesity and the development of various noncommunicable diseases. However, large animal models for studying obesity-related meta-inflammation are lacking. Minipigs have great potential as models for human diseases, warranting investigation of the performance of the Göttingen minipig as a model for obesity-associated meta-inflammation. Here, we fed 26 pigs a high-fat, fructose and cholesterol diet (HFFC) or a standard diet (SD) for 103 days, resulting in the HFFC group having a 45% higher body weight and 16% larger abdominal circumference by the end of the experiment. Meta-inflammation was shown in the HFFC group by elevated serum concentrations of the acute phase protein C-reactive protein for more than 60 days during development of obesity, accompanied by increased numbers of circulating neutrophils and monocytes. Additional obesity-related abnormalities included dyslipidemia, hepatosteatosis and transcriptional changes to genes related to inflammation and metabolism in circulating leukocytes, liver and visceral adipose tissue. Notably, the transcription of genes related to lipid metabolism, namely ATP-binding cassette subfamily A member 1 (*ABCA1*) and ATP-binding cassette subfamily G member 1 (*ABCG1*), was elevated in liver, visceral adipose tissue and circulating leukocytes (*ABCA1* only) in the HFFC group compared with the SD group. The development of obesity was accompanied by endotoxemia, indicated by a 2.5-fold increase in serum lipopolysaccharide concentration in the HFFC group compared with the SD group, suggesting increased intestinal permeability. In conclusion, the described Göttingen minipig model convincingly links diet-induced obesity, meta-inflammation and endotoxemia, achieved by short-duration HFFC dieting.

## Abstract (web summary)

The Article describes a minipig model of obesity-associated meta-inflammation, characterized by increased C-reactive protein levels and white blood cells, together with changes in the expression of genes related to inflammation and metabolism.

## Main

Chronic low-grade systemic inflammation associated with metabolic disorders, known as meta-inflammation^1^, is a common feature of obesity associated with a range of noncommunicable diseases, including cardiovascular diseases, type 2 diabetes, chronic respiratory diseases and cancers^2^. Prominent risk factors for these conditions include abdominal obesity and elevated levels of triglycerides, both key criterions of metabolic syndrome^3^. Furthermore, meta-inflammation increases the risk of severe infectious diseases, including COVID-19 and influenza^4^. The underlying mechanisms of how meta-inflammation develops and how it is linked to disease severity are not fully understood, stressing the need for better, highly translational animal models reproducing obesity-driven meta-inflammation.

Meta-inflammation can be characterized by a persistent increase in circulating levels of the acute-phase protein (APP) C-reactive protein (CRP) and white blood cells such as neutrophils^1,5–7^. Meta-inflammation can be caused by high intake of fat, cholesterol and sugars, leading to adipocyte hypertrophy, which in turn triggers immune cell infiltration and the secretion of pro-inflammatory adipokines^1,5^. In addition, ectopic lipid accumulation in nonadipose tissues, such as the liver, can potentially lead to lipotoxicity^8,9^. Furthermore, energy-dense diets can result in intestinal dysbiosis, which may increase intestinal permeability, allowing the leakage of endotoxins, predominantly lipopolysaccharides (LPS), into systemic circulation, causing endotoxemia^10^. The passage of these harmful substances into the bloodstream, known as ‘leaky gut’ syndrome, can trigger chronic inflammation^10^. However, the causal relation between obesity, leaky gut and meta-inflammation remains poorly understood.

Large animal models, such as the pig, show high translational relevance in the study of complex diseases. This includes the multifactorial condition of human obesity, associated meta-inflammation and obesity-related comorbidities^11–15^. The high translational value of pig models is rooted in their similar metabolism, gastrointestinal tract, cardiovascular system and proportionally similar organ size compared with humans^13,16^. Pigs are prone to obesity and obesity-related comorbidities, and the Göttingen minipig is frequently used in biomedical research on obesity, metabolic syndrome and coronary vascular disease such as atherosclerosis. However, meta-inflammation associated with diet-induced obesity is not well characterized in porcine models. Although obesity can be induced in Göttingen minipigs by ad libitum feeding on a standard diet (SD)^17^, this approach has not resulted in meta-inflammation^18,19^. In some studies, feeding a more energy-dense diet for >5 months resulted in elevated levels of pro-inflammatory cytokines and APPs in minipig models^20–22^. However, none of these studies reported the levels of these and other inflammatory markers in circulation continuously throughout the development of diet-induced obesity.

The aim of the present study was to establish, characterize and evaluate the Göttingen minipig as a novel large animal model for diet-induced meta-inflammation using a Western-like diet high in fat, fructose and cholesterol. This study demonstrates long-term increased CRP and LPS concentrations in circulation, accompanied by changes in the expression of relevant genes in circulating leukocytes during the development of obesity, and in the liver and visceral adipose tissue (VAT) at termination, suggesting a chronic systemic inflammatory state in this large animal model. These findings support the use of pigs as the state-of-the-art model for human obesity and its comorbidities.

## Results

### Steady increase in body weight and abdominal body circumference during the dieting period

**Fig. 1:**
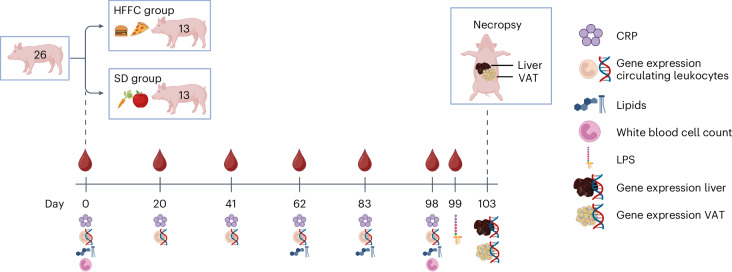
Experimental setup. Twenty-six Göttingen minipigs were divided into two equal groups and fed either the HFFC diet (*n* = 13) or the SD diet (*n* = 13) for 103 days. Blood samples were collected from all animals throughout the study. CRP concentrations were quantified on days 0, 20, 41, 62, 83 and 98. Lipid concentrations were quantified on days 0, 62, 83 and 98. White blood cell counts were performed on days 0 and 98. LPS concentrations were quantified on day 99. Gene expression in circulating leukocytes was quantified on days 0, 20, 41, 62, 83 and 98. Gene expression in liver tissue and VAT was quantified in four randomly selected pigs from each diet group on day 103. Created in BioRender. Henriksen, B. (2025) https://BioRender.com/25rsuz8.

Twenty-six castrated male Göttingen minipigs, 9–10 weeks of age, were divided into two groups. One group received a diet high in fat, fructose and cholesterol (HFFC; *n* = 13), and the other group received a SD (*n* = 13) for 103 days (Fig. 1).

**Fig. 2:**
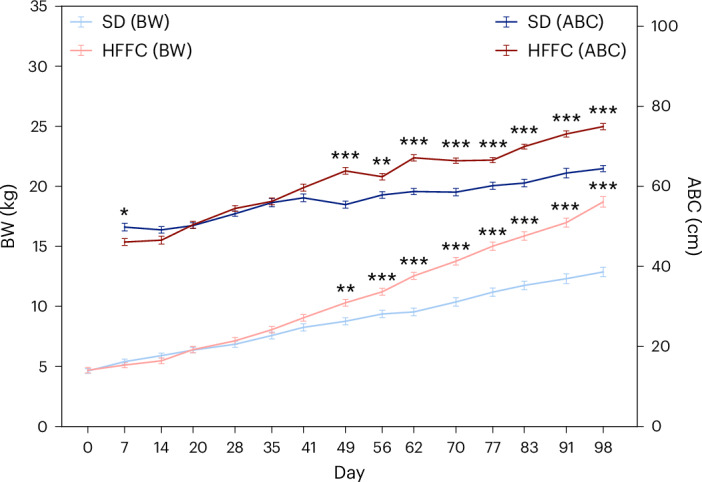
The HFFC diet induces an increase in BW and ABC in Göttingen minipigs. From day 49 of the study, significantly increased BWs (left *y* axis) and ABC (right *y* axis) were observed in the HFFC group compared with the SD group. Statistical significance of differences between the HFFC (*n* = 13) and SD (*n* = 13) groups was determined with Welch’s *t*-test and Benjamini–Hochberg corrections (*P < 0.05, **P < 0.01, ***P < 0.001). Error bars show the s.e.m.

Body weight (BW) and abdominal circumference (ABC) increased steadily in both groups; however, the increase was accelerated in the HFFC group (Fig. 2), demonstrating an obesogenic effect of the HFFC diet in Göttingen minipigs. From day 49 days and onward, both BW and ABC were significantly higher in the HFFC group compared with the SD group (BW: *P* = 0.002; ABC: *P* = 8.5 × 10^−7^, two-sided Welch’s *t*-tests with Benjamini–Hochberg corrections) (Fig. 2). After 98 days, the average BW and ABC in the HFFC group were 45% and 16% higher than in the SD group, respectively. A slightly but significantly (*P* = 0.014) lower ABC was initially observed in the HFFC group as the pigs adjusted to the new diet.

### The HFFC diet induces meta-inflammation in Göttingen minipigs

**Fig. 3:**
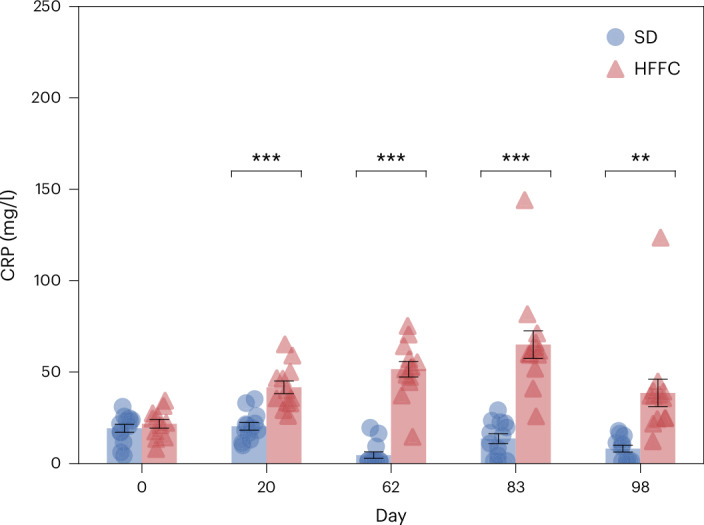
The HFFC diet is associated with increased CRP serum concentrations in Göttingen minipigs. Statistical significance of differences between the HFFC (*n* = 13) and SD (*n* = 13) groups was determined with Welch’s *t*-test and Benjamini–Hochberg corrections (**P < 0.01, ***P < 0.001). The bars and error bars represent the mean and s.e.m.

The average serum concentration of classical inflammation biomarker CRP was significantly (*P* = 8.6 × 10^−5^, two-sided Welch’s *t*-tests with Benjamini–Hochberg corrections) increased in the HFFC group compared with the SD group already from day 20 and remained higher in this group for the rest of the experiment. On day 20, the average serum concentration of CRP was twice as high in the HFFC group compared with the SD group, and between day 62 and day 98, the difference ranged from approximately 5-fold to 11-fold (Fig. 3). Technical issues with CRP quantification in samples from day 41 made results from this time point unreliable, and they were therefore discarded.

**Fig. 4:**
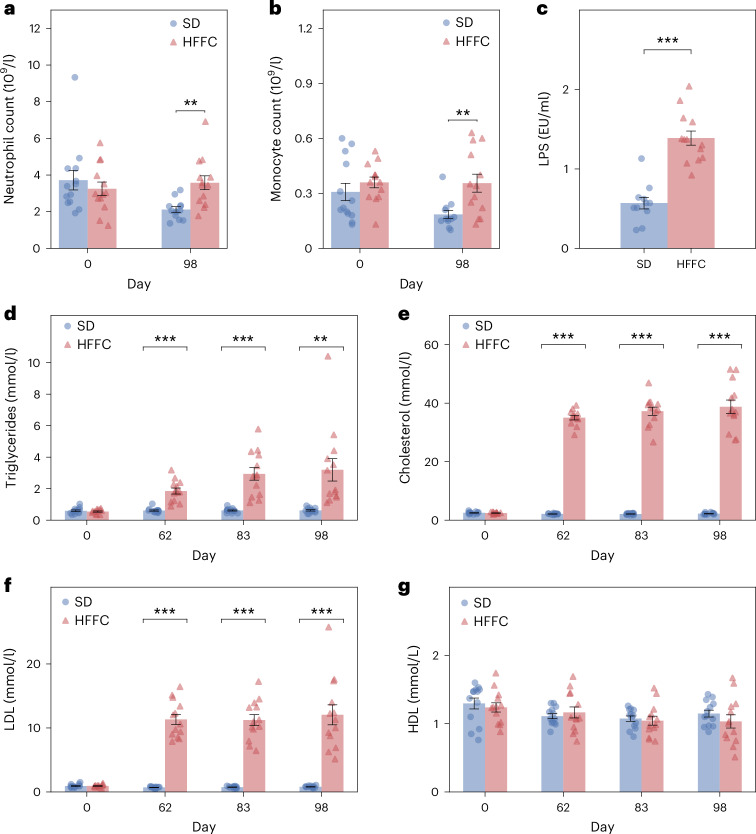
The HFFC diet induces changes to circulating leukocytes and leads to endotoxemia and dyslipidemia. **a**, Circulating neutrophil counts. **b**, Circulating monocyte counts. **c**, Serum LPS concentration (in endotoxin units (EU)) on day 99 after initiation of diet regimes. **d**, Circulating triglyceride concentrations. **e**, Circulating total cholesterol concentration. **f**, Circulating LDL concentrations. **g**, Circulating HDL concentrations. Statistical significance of differences between cell counts and lipid concentrations in the HFFC (*n* = 13) and SD (*n* = 13) group was determined using Welch’s *t*-test with Benjamini–Hochberg corrections applied for multiple testing (**P < 0.01, ***P < 0.001). Bars and error bars show the mean and s.e.m.

No difference in the number of circulating immune cells was observed between the dieting groups before initiation of the dieting regime; however, on day 98, the number of circulating neutrophils and monocytes in the HFFC group was approximately twice as high compared with the SD group (Fig. 4a,b).

To identify a possible cause of meta-inflammation, the serum concentration of LPS was measured on day 99. The serum LPS concentration was found to be significantly (*P* = 4.4 × 10^−7^, two-sided Welch’s *t*-tests with Benjamini–Hochberg corrections) elevated in the HFFC group, almost 2.5 times higher than in the SD group (Fig. 4c). Furthermore, a significant (*P* = 0.02, Spearman’s correlation coefficient) positive correlation was observed between serum CRP and LPS concentrations (Table 1).

**Table 1:** Correlation between CRP concentration and anthropometric and biochemical parameters

	CRP concentration											
	Day 0		Day 20		Day 62		Day 83		Day 98		Overall	
	*r*_S_	*P*	*r*_S_	*P*	*r*_S_	*P*	*r*_S_	*P*	*r*_S_	*P*	*r*_S_	*P*
**BW**	0.38*	0.04	−0.08	0.67	0.71*	6.04 × 10^−6^	0.71*	5.62 × 10^−6^	0.73*	2.75 × 10^−6^	0.16*	0.03
**ABC**	–	–	−0.02	0.93	0.80*	3.44 × 10^−8^	0.75*	8.59 × 10^−7^	0.65*	6.61 × 10^−5^	0.38*	1.10 × 10^−6^
**LPS conc**.	–	–	–	–	–	–	–	–	–	–	0.48*	0.02
**Triglyceride conc**.	0.11	0.62	–	–	0.70*	7.45 × 10^−5^	0.62*	7.39 × 10^−4^	0.61*	9.34 × 10^−4^	0.57*	2.77 × 10^−10^
**Total cholesterol conc**.	0.51*	0.01	–	–	0.77*	5.80 × 10^−6^	0.70*	7.74 × 10^−5^	0.70*	5.98 × 10^−5^	0.76*	6.09 × 10^−20^
**LDL conc**.	0.17	0.43	–	–	0.66*	2.24 × 10^−4^	0.68*	1.29 × 10^−4^	0.75*	1.10 × 10^−5^	0.70*	4.64 × 10^−16^
**HDL conc**.	0.36	0.09	–	–	0.09	0.66	−0.32	0.11	−0.25	0.21	−0.08	0.44

Statistically significant Spearman’s correlations (*r*_S_) are indicated by an asterisk. *P*, *P* value; conc., concentration.

The HFFC diet induced dyslipidemia, indicated by significantly higher concentrations of triglycerides (day 62: *P* = 8.8 × 10^−5^; day 83: *P* = 0.0002; day 98: *P* = 0.005, two-sided Welch’s *t*-tests with Benjamini–Hochberg correction), total cholesterol (day 62: *P* = 6.1 × 10^−13^; day 83: *P* = 1.4 × 10^−11^; day 98: *P* = 2.5 × 10^−9^) and low-density lipoprotein (LDL) (day 62: *P* = 5.8 × 10^−8^; day 83: *P* = 8.5 × 10^−8^; day 98: *P* = 1.5 × 10^−5^) in the HFFC group compared with the SD group at all sampled time points after the diet period was initiated (days 62, 83 and 98) (Fig. 4d–f), whereas high-density lipoprotein (HDL) concentrations remained unchanged in both groups throughout the study (Fig. 4g). From day 62 onward, a significant positive correlation was observed between CRP and total cholesterol concentrations (day 62: *P* = 5.8 × 10^−6^; day 83: *P* = 7.7 × 10^−5^; day 98: *P* = 6.0 × 10^−5^; Spearman’s rank correlation), between CRP and LDL concentrations (day 62: *P* = 2.2 × 10^−4^; day 83: *P* = 1.3 × 10^−4^; day 98: *P* = 1.1 × 10^−5^), as well as between CRP and triglyceride concentrations (day 62: *P* = 7.5 × 10^−5^; day 83: *P* = 7.4 × 10^−4^; day 98: *P* = 9.3 × 10^−4^) (Table 1). Significant positive correlations were also found between CRP concentration and BW (day 62: *P* = 6.0 × 10^−6^; day 83: *P* = 5.6 × 10^−6^; day 98: *P* = 2.8 × 10^−6^), as well as between CRP concentration and ABC (day 62: *P* = 3.4 × 10^−8^; day 83: *P* = 8.6 × 10^−7^; day 98: *P* = 6.6 × 10^−5^), from day 62 onward (Table 1).

### The HFFC diet induces changes to gene expression in VAT and circulating leukocytes

Gene expression analysis and histopathological examination of VAT was performed on four randomly selected animals from each of the two diet groups at termination of the experiment. Gene expression results for all genes quantified in VAT can be found in Supplementary Table 1, while results for selected genes are presented in Table 2. In line with the observed dyslipidemia in the HFFC group, two genes encoding proteins associated with uptake of lipids and cholesterol, ATP-binding cassette subfamily A member 1 (*ABCA1*) and ATP-binding cassette subfamily G member 1 (*ABCG1*), were upregulated in the VAT of the HFFC group compared with the SD group, as was the expression of the appetite regulator leptin (*LEP*) (Table 2). Important genes associated with inflammation were upregulated in VAT in the HFFC group including interleukin (IL)-1α (*IL1A*), interferon-γ (*IFNG*) and the positive APPs orosomucoid 1 (*ORM1*) and lactotransferrin (*LTF*), whereas the gene expression of pro-inflammatory cytokines *IL6* and tumor necrosis factor (*TNF*) was not affected by the HFFC diet (Table 2). Transthyretin (*TTR*), recognized as a negative APP, and the anti-inflammatory interleukin 1 receptor antagonist (*IL1RN*) were downregulated in the HFFC group relative to the SD group, further indicating an altered inflammatory environment in the VAT of the HFFC group. In contrast to the expectations, the conventionally negative APP albumin (*ALB*), was more highly expressed in the HFFC group than in the SD group. Consistent with its role as a positive APP and the elevated concentrations of LPS in circulation, LPS-binding protein (*LBP*) was also upregulated in the HFFC group. In agreement with histopathological examination of VAT from pigs in the HFFC group (data not shown), which revealed no sign of neutrophil or monocyte infiltration, there was no observed increase in the gene expression of relevant cellular markers (cluster of differentiation (*CD*) *68*, *CD163*, *CD80* and *CD86*)).

**Table 2:** Gene expression in VAT

Gene	VAT relative expression level ± s.e.m.	
	SD group	HFFC group
*LTF*	1 ± 0.3	9.9 ± 2.8
*ABCA1*	1 ± 0.2	4.7 ± 1.0
*LEP*	1 ± 0.2	3.2 ± 0.8
*ABCG1*	1 ± 0.2	2.9 ± 0.6
*LBP*	1 ± 0.5	2.6 ± 0.3
*IL1A*	1 ± 0.2	2.6 ± 0.3
*IFNG*	1 ± 0.2	2.3 ± 0.3
*ORM1*	1 ± 0.3	2.2 ± 0.5
*ALB*	1 ± 0.1	2.0 ± 0.4
*CD80*	1 ± 0.3	1.4 ± 0.3
*CXCL8*	1 ± 0.2	1.2 ± 0.3
*CD68*	1 ± 0.4	1.2 ± 0.1
*TNF*	1 ± 0.2	1.1 ± 0.5
*IL6*	1 ± 0.2	1.1 ± 0.2
*CD163*	1 ± 0.4	1.1 ± 0.2
*SAA3*	1 ± 0.3	0.9 ± 0.4
*CD86*	1 ± 0.4	0.8 ± 0.1
*IL1RN*	1 ± 0.4	0.5 ± 0.1
*CXCL14*	1 ± 0.2	0.5 ± 0.0
*TTR*	1 ± 0.9	0.2 ± 0.1

Mean gene expression in the SD group (*n* = 3) is scaled to 1, and mean gene expression in the HFFC group (*n* = 4) is shown relative to the SD group. Results are presented as group means ± s.e.m.

**Fig. 5:**
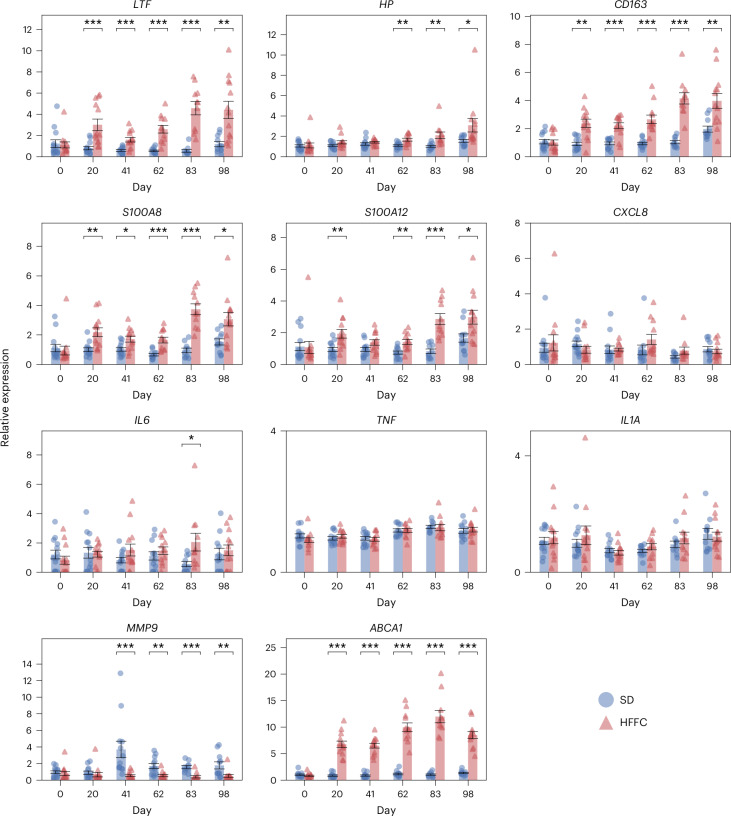
The HFFC diet induces changes in gene expression in circulating leukocytes throughout the dieting period. The expression of each gene is scaled to the expression level in the SD group on day 0. Statistical significance of differences between the HFFC (*n* = 13) and SD (*n* = 13) groups at individual time points was determined with Welch’s *t*-test and Benjamini–Hochberg corrections (*P < 0.05, **P < 0.01, ***P < 0.001). Bars and error bars show the mean and s.e.m.

Analysis of gene expression in circulating leukocytes revealed that the HFFC diet resulted in significant upregulation of the inflammation-related genes *LTF* (day 20: *P* = 0.0004; day 41: *P* = 0.007; day 62: *P* = 4.8 × 10^−6^; day 83: *P* = 1.4 × 10^−6^; day 98: *P* = 0.001, two-sided Welch’s *t*-tests on log_2_-transformed data with Benjamini–Hochberg correction), haptoglobin (*HP*) (day 62: *P* = 0.009; day 83: *P* = 1.4 × 10^−6^; day 98: *P* = 0.001), *CD163* (day 20: *P* = 0.03; day 41: *P* = 0.0007; day 62: *P* = 4.79 × 10^−6^; day 83: *P* = 5.02 × 10^−7^; day 98: *P* = 0.005), and S100 calcium-binding proteins A8 (*S100A8*) (day 20: *P* = 0.003; day 41: *P* = 0.03; day 62: *P* = 0.0003; day 83: *P* = 0.0007; day 98: *P* = 0.01) and A12 (*S100A12*) (day 20: *P* = 0.008; day 62: *P* = 0.009; day 83: *P* = 0.0003; day 98: *P* = 0.02) at most or all sampled time points after initiation of the dieting regime compared with the SD diet (Fig. 5). The classical pro-inflammatory cytokine *IL6* was significantly upregulated in the HFFC group only on day 83 (*P* = 0.01), whereas expression of *TNF*, *IL1A* and C-X-C motif chemokine ligand 8 (*CXCL8*) was not different between the two diet groups in circulating leukocytes at any time point (Fig. 5). In addition, the gene expression levels of the inflammatory matrix metallopeptidase 9 (*MMP9*) were significantly reduced in circulating leukocytes of the HFFC group compared with the SD group from day 41 onward (day 41: *P* = 4.75 × 10^−5^; day 62: *P* = 0.003; day 83: *P* = 0.0002; day 98: *P* = 0.005) (Fig. 5). Similar to VAT, expression of *ABCA1* was significantly upregulated in circulating leukocytes in the HFFC group from day 20 onward (day 20: *P* = 9.56 × 10^−11^; day 41: *P* = 9.56 × 10^−11^; day 62: *P* = 9.56 × 10^−11^; day 83: *P* = 4.33 × 10^−6^; day 98: *P* = 1.85 × 10^−11^) (Fig. 5). Gene expression results for all genes quantified in circulating leukocytes can be found in Supplementary Table 2.

### The HFFC diet is associated with steatosis and differential gene expression in the liver

**Fig. 6:**
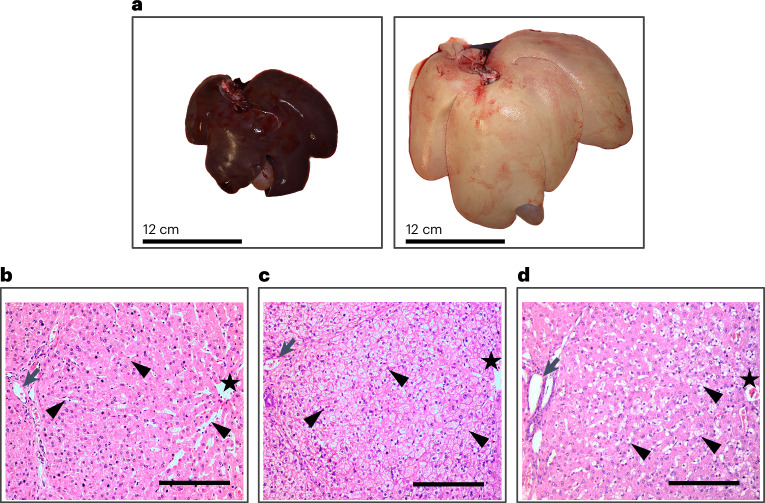
Macroscopic and microscopic changes observed in livers of HFFC-fed pigs. **a**, Livers from pigs in the HFFC group displayed steatosis (right), while livers from pigs in the SD group were normal (left). Histopathological examination was performed on pigs from both the HFFC (*n* = 4) and SD (*n* = 4) groups. **b**, Histological analysis of the liver from an SD-fed pig. Arrowheads, sinusoidal space; star, central vein; arrow, portal arteriole. **c**, Liver from HFFC-fed pig. Arrowheads, microvesicular lipids; star, central vein; arrow, portal arteriole. **d**, Liver from HFFC-fed pig. Arrowheads, large lipid vacuoles; star, central vein; arrow: portal arteriole. Scale bars, 200 µm (**b**–**d**).

Livers in all HFFC-fed pigs were pale and enlarged compared with livers in the SD-fed pigs (Fig. 6a). Histopathological examination revealed that livers of the SD-fed pigs showed normal parenchyma characterized by polygonal hepatocytes and visible sinusoidal spaces (Fig. 6b). A few hepatocytes with lipid vacuoles were seen in two out of four SD-fed pigs. In the HFFC-fed pigs, parenchyma was characterized by severe steatosis with swollen hepatocytes showing decreased stainability of the cytoplasm due to microvesicular lipids (Fig. 6c). In addition, large lipid droplet vacuolation was found in one of four livers (Fig. 6d). The increased size of the hepatocytes made it impossible to recognize the polygonal appearance and the contour of the sinusoidal space in the four HFFD animals. No immune cell infiltration was observed in either diet group, suggesting absence of inflammation in the liver.

Gene expression results for all genes quantified in the liver can be found in Supplementary Table 3. Gene expression results for selected genes are presented in Table 3. Surprisingly, the expression of *CRP* and other APPs including *HP* and *ORM1*, was not upregulated in the livers of HFFC-fed pigs but rather tended to be downregulated compared with SD-fed pigs (Table 3). However, several other genes involved in tissue inflammation, including serum amyloid A3 (*SAA3*), *LTF*, *TNF*, *IL1A*, *IFNG*, *S100A8*, *S100A12*, *MMP9*, C-C motif chemokine ligand 3 and 4 (*CCL3* and *CCL4*), *CXCL8*, *CXCL10*, *CXCL14*, secreted phosphoprotein 1 (*SPP1*) and C-X-C motif chemokine receptor 4 (*CXCR4*), were expressed at higher levels in the livers of the HFFC group compared with the SD group (Table 3). In contrast to expression patterns observed in the VAT, the genes encoding the dendritic cell, monocyte and macrophage markers *CD68*, *CD80* and *CD86* were upregulated in livers of the HFFC group, whereas the expression of the tissue-resident macrophage marker *CD163* was unchanged in the HFFC-fed pigs compared with the SD-fed pigs. In agreement with the expression signatures in VAT, *ABCA1* and *ABCG1* were upregulated in livers of the HFFC group compared with the SD group, which was also the case for other genes involved in fatty acid metabolism associated with high uptake of lipids and cholesterol, such as *CD36*, peroxisome proliferator activated receptor gamma (*PPARG*) and stearoyl-CoA desaturase (*SCD*), the latter of which was downregulated in VAT of the HFFC group. By contrast, hepatic expression of the fatty acid synthase gene *FASN* tended to be downregulated. Klotho beta (*KLB*), encoding β-klotho, serving as a co-receptor for fibroblast growth factor 21 involved in energy homeostasis, adipogenesis and bile acid production, was the most strongly downregulated gene in the livers of the HFFC group.

**Table 3:** Gene expression in the liver

Gene	Liver relative expression level ± s.e.m.	
	SD group	HFFC group
*CXCL14*	1 ± 0.1	113.6 ± 25.8
*PPARG*	1 ± 0.3	53.7 ± 11.5
*ABCG1*	1 ± 0.2	35.8 ± 4.0
*SAA3*	1 ± 0.4	27.8 ± 13.4
*MMP9*	1 ± 0.5	20.2 ± 11.3
*SPP1*	1 ± 0.2	13.0 ± 2.3
*CD36*	1 ± 0.2	7.7 ± 2.2
*CD68*	1 ± 0.1	7.3 ± 1.7
*CXCL8*	1 ± 0.2	6.6 ± 1.9
*S100A8*	1 ± 0.3	6.3 ± 0.8
*CXCL10*	1 ± 0.4	6.2 ± 2.4
*CXCR4*	1 ± 0.2	6.0 ± 1.8
*CD86*	1 ± 0.1	5.6 ± 0.5
*LTF*	1 ± 0.1	4.0 ± 1.1
*CCL3*	1 ± 0.2	3.8 ± 0.4
*S100A12*	1 ± 0.2	3.8 ± 0.5
*CD80*	1 ± 0.2	3.4 ± 0.8
*NOS2*	1 ± 0.3	3.2 ± 0.7
*IFNG*	1 ± 0.3	3.0 ± 0.6
*ABCA1*	1 ± 0.3	2.8 ± 0.3
*TNF*	1 ± 0.1	2.5 ± 0.5
*SCD*	1 ± 0.1	2.4 ± 0.4
*CCL4*	1 ± 0.1	2.2 ± 0.4
*IL1A*	1 ± 0.1	2.2 ± 1.0
*HP*	1 ± 0.2	0.5 ± 0.2
*ORM1*	1 ± 0.2	0.5 ± 0.04
*FASN*	1 ± 0.2	0.3 ± 0.1
*KLB*	1 ± 0.3	0.009 ± 0.004

Mean gene expression in the SD group (*n* = 4) is scaled to 1, and mean gene expression in the HFFC group (*n* = 4) is shown relative to the SD group. Results are presented as group means ± s.e.m.

## Discussion

We successfully induced a state of obesity and meta-inflammation in castrated male Göttingen minipigs by feeding them a Western-like HFFC diet. The presence of meta-inflammation was confirmed by consistently elevated serum CRP concentrations from day 20 and substantiated by increased counts of circulating neutrophils and monocytes on day 98 compared with pigs fed the SD diet. This study continuously monitored and demonstrated long-term diet-induced increases in serum CRP concentrations, along with elevated serum LPS concentrations and increased expression of genes related to inflammation and metabolism in the VAT and liver. These findings align well with those observed in children and adults with obesity^6,7,23–31^ who are at increased risk of diseases such as cardiovascular diseases and type 2 diabetes^32,33^. The presence of meta-inflammation was indicated by differential expression of several genes such as *LTF*, *S100A8* and *S100A12*, associated with inflammation, and *ABCA1*, associated with lipid metabolism, not only in circulating leukocytes but also in VAT, liver tissue or both, in the obese HFFC-fed pigs compared with SD-fed pigs. Future validation of transcriptional results by protein quantification would further substantiate our findings.

Several studies have used minipigs as obesity models using various HFFC-like diets to mimic a Western-like diet or simply using ad libitum feeding with a SD^18,20,34–40^. While obesity was achieved in all studies, the accompanying effects varied: some animals displayed dyslipidemia^34,38^, elevated serum CRP concentration or other inflammatory markers^20–22,34,41^, or varying degrees of hepatic inflammation^20–22,34,38,39^. The variations in these different studies may be attributed to differences in diet composition, pig breed and study duration. Sex may also affect the outcome, as it has been found to influence the ability to develop obesity, as has the sterilization status of the animal^17,35^. To avoid this confounder, we used only castrated males; however, future studies would benefit from including female pigs as well. Although the Göttingen minipig is well characterized and widely used in studies of obesity and metabolic syndrome and has shown high translational value for these conditions in humans, the use of the Ossabaw minipig model might also be considered in future studies, as the model may produce results with even higher fidelity^12,14^.

In the present study, all the responses commonly exhibited by obese individuals were observed. Given the importance of diet composition and study duration for inducing meta-inflammation, continuous measurements of inflammatory biomarkers, as performed in the present study, can enhance our understanding of the causality, dynamics and the potential development of tolerance to long-term inflammation and exposure to Western-like diets.

In the present study, serum CRP concentrations were significantly higher in obese pigs compared with nonobese pigs from day 20, even though we observed no increase in expression of *CRP* in liver and VAT at the end of the study. Other studies have previously demonstrated that administering similar HFFC diets to minipigs for extended periods of time did not result in systemic inflammation at the end of the study despite the pigs developing obesity^22,38^. In these studies, the duration of dieting was longer (24 weeks or 13 months, respectively) than the 14-week diet period of the present study. In addition, changes to CRP concentrations or other inflammatory markers were not continuously monitored in these two studies. In our HFFC group, CRP concentration continuously increased from day 0 to day 83, followed by a decline from day 83 to day 98, although it still remained significantly increased in the HFFC group compared with the SD group. This finding, taken together with the unchanged liver expression of *CRP*, may suggest that concentrations of circulating CRP in obese pigs could be leveling off toward the end of our experiment. These findings add a level of complexity to the understanding of underlying mechanisms of obesity, meta-inflammation and noncommunicable diseases.

Although obesity is commonly thought to induce inflammation, we observed an increase in serum CRP concentration in the HFFC group 4 weeks before significant differences in BW were observed between the two diet groups. Several studies have suggested that obesity alone does not necessarily lead to increased concentrations of inflammatory markers in circulation^18,19,22^. The present findings suggest that the diet, rather than obesity alone, may be an important contributor to the observed inflammatory state. Accordingly, no correlation was seen between CRP concentrations and BW or ABC on day 20, when CRP was already significantly elevated in the HFFC group. In addition to the evidence of inflammation provided by CRP concentrations, the significant increase in gene expression of inflammatory markers such as *LTF*, *HP*, *S100A8* and *S100A12* in leukocytes, already from day 20, emphasizes that a state of inflammation might precede the onset of significant weight gain.

Meta-inflammation associated with obesity is considered to be of multifactorial origin. Although adipose tissue is often considered a key contributor, studies have suggested that bacterial components including LPS from a leaky gut may also have a role in inducing and sustaining systemic inflammation^10,42^. Consumption of energy dense Western-like diets might result not only in adipose tissue expansion and dyslipidemia but also in increased concentrations of circulating LPS due to gut hyperpermeability, potentially related to microbiota dysbiosis that adversely affects gut barrier integrity^43^. Bacterial LPS may thus enter the bloodstream, contributing to systemic inflammation and potentially to metabolic changes, as observed both in the present study and in human studies^42,44,45^. In the present study, LPS concentration in the serum from the HFFC pigs was almost 2.5 times higher than that in SD pigs, mirroring findings in obese mice and humans^42,46^. Moreover, we observed a significant positive correlation between serum LPS and CRP concentrations, suggesting a potential role of endotoxemia in diet-induced meta-inflammation. However, as serum LPS concentrations were measured only at the end of the experiment, it can only be speculated whether elevated LPS concentrations also preceded the onset of obesity and thereby contributed to the early increase in serum CRP concentrations in the HFFC group. Whereas this would be in agreement with the elevated expression of the abovementioned inflammatory markers in circulating leukocytes throughout the experiment (for example, LTF and S100A8), we found no or limited increase in expression of pro-inflammatory genes *IL1A*, *IL6*, *TNF* and *CXCL8* in leukocytes. Along with endotoxemia, dyslipidemia also showed a significant association with inflammation in our model. We observed consistent positive correlations between CRP concentrations and lipid parameters from day 62 onward, even as CRP concentrations declined. These associations suggest that both metabolic disturbances and endotoxemia may contribute to the inflammatory state in diet-induced obesity.

Adipocyte hypertrophy and metabolic changes may lead to adipose tissue inflammation, causing adipocytes to secrete pro-inflammatory adipokines and chemokines. We observed that several genes centrally associated with inflammation, including *IL1A*, *IFNG* and APPs such as *LTF*, *ORM1* and *ALB*, contributed to an inflammatory gene expression signature in VAT of the obese pigs in the present study. Surprisingly, the expression of inflammatory cytokines such as *TNF*, *IL6* and *CXCL8* and the APP *SAA3* remained unchanged, resembling our observations in circulating leukocytes. In addition, histological examination of VAT from obese pigs showed no lymphocyte infiltration, and gene expression analysis indicated an unchanged expression of inflammatory cytokines and immune cell surface proteins, including *CD68*, *CD80* and *CD86*, with downregulation of the monocyte attractant chemokine *CXCL14*. This finding contrasts with human studies, which have reported increased expression of *TNF* and *IL6* in the adipose tissue of obese individuals^43,44,47^. However, our observations align with a study using Göttingen minipigs fed a similar HFFC diet for 13 months, where no upregulation of inflammatory cytokines in VAT and no monocyte infiltration in the adipose tissues were observed^38,40^. We found evidence in VAT of gene expression that may contribute to an anti-inflammatory effect in obese pigs, namely the upregulation of *ABCA1* and *ABCG1*, indicating diet-induced metabolic changes. ABCA1 and ABCG1 promote cholesterol and lipid efflux, mitigating inflammatory effects associated with obesity by maintaining the levels of anti-inflammatory HDL particles^48,49^. Our findings are consistent with findings from human and pig studies, showing increased mRNA levels of *ABCA1* and/or *ABCG1* in adipose tissue of obese participants^40,49^. Expression of *ABCA1* and *ABCG1* might have a role in balancing pro-inflammatory and anti-inflammatory processes during chronic inflammation, highlighting the interplay of metabolic and inflammatory pathways in achieving immune homeostasis in adipose tissue.

The HFFC diet led to steatosis of the liver. Alterations to the hepatic lipid metabolism were evident from the increased expression of related genes, including *ABCA1*, *ABCG1*, *PPARG* and *CD36*, whereas *FASN* was downregulated in obese pigs. *KLB*, associated with fatty acid oxidation and suppression of lipogenesis, was strongly downregulated in the livers of obese pigs in concordance with other studies^35,40^. The reduction in *KLB* expression could enhance lipogenesis, potentially exacerbating lipotoxicity and inflammation within hepatocytes^50^, as well as promote the observed development of liver steatosis in obese pigs^51^. Transcriptionally, the steatotic livers also showed indications of increased inflammation, with elevated expression of *TNF*, *CXCL8*, *CXCL10*, *CXCL14*, *CXCR4*, *S100A8*, *S100A12*, *MMP9*, *SAA3* and *SPP1*, all of which are involved in immune cell recruitment and infiltration. This is relevant as the association between liver steatosis and an inflammatory hepatic environment is well described^52^.

In conclusion, we successfully established a minipig model for diet-induced obesity with accompanying meta-inflammation and endotoxemia, offering a valuable tool to enhance our understanding of the mechanisms linking obesity to its serious comorbidities. Meta-inflammation was confirmed through persistently elevated CRP serum concentrations, dyslipidemia, endotoxemia and increased circulating numbers of neutrophils and monocytes, along with upregulated expression of genes related to inflammation and metabolism in circulating leukocytes, the liver and adipose tissue. These findings further underline the multifactorial nature of diet-induced meta-inflammation. Beyond elucidating the connections between obesity, metabolic syndrome and cardiometabolic diseases, this animal model holds substantial relevance for exploring the role of meta-inflammation in exacerbating infectious diseases such as influenza and COVID-19. The model may also guide the development of improved therapeutics and vaccines against such infections in high-risk populations in the future.

## Methods

### Animals and diets

The study was approved by the Animal Experiments Inspectorate, Ministry of Justice, Denmark (permit number 2016-15-0201-01022). Nine-week-old, castrated male Göttingen minipigs (*n* = 26, based on statistical power calculation) (Ellegaard Göttingen Minipigs A/S) were housed on a 12-h light/12-h dark cycle with free access to water and were fed twice daily. Animals were randomized into two dietary groups (Fig. 1): a control group fed a standard minipig chow (Mini-pig, Special Diets Services, Supplementary Table 4) according to the breeder’s guidelines (SD group, *n* = 13), and a group fed a diet high in fat, fructose and cholesterol (20,045 ppm) (Ossabaw pig diet – 5B4L, TestDiet, Supplementary Table 4) (HFFC group, *n* = 13). The fat component of the HFFC diet was made up of 52.9% saturated fatty acids, 25.5% monounsaturated fatty acids, 8.5% polyunsaturated fatty acids and 13.1% other (by weight). Drinking water was freely available. The HFFC group was fed 2.5–3.0% of the average BW of the group and thus adjusted according to the weekly weighing. All animals were weighed weekly, and the ABC was measured at the umbilicus. Blood samples were collected on days 0, 20, 41, 62, 83, 98 and 99. On day 103, the animals were sedated by Zoletil and euthanized by intracardiac injection of pentobarbital. The animals were fasted for a minimum of 12 h before blood collection. Liver and VAT samples were collected for transcriptional analysis from eight randomly selected pigs (four from the SD group and four from the HFFC group).

### Sampling and handling of blood and tissue samples

Blood samples were collected in PAXgene Blood RNA Tubes (PreAnalytiX), BD Vacutainer K_3_EDTA Tubes (BD Company) and BD Vacutainer Serum Tubes coated with silica (BD Company) at the time points indicated in Fig. 1. After blood draw, tubes were inverted eight to ten times. PAXgene Blood RNA Tubes were stored at room temperature in an upright position for at least 2 h before storage at −20 °C until RNA extraction. K_3_EDTA-coated tubes were stored on ice for 4–6 h and at 4 °C until cell counting the following day. Serum tubes were stored at room temperature for 4–6 h before centrifugation at 1,200*g* for 10 min. Liver and VAT samples were collected at the end of the experiment and stored overnight in RNAlater (Thermo Fisher Scientific) at 4 °C before transferring to −20 °C until RNA extraction.

### CRP detection by ELISA

CRP serum concentration was determined by dendrimer-coupled cytidine diphosphocholine indirect ELISA as described previously^53^. The lower limit of detection was 1.42 mg/l.

### Biochemical analysis and white blood cell count

Plasma concentrations of total cholesterol, LDL cholesterol, HDL cholesterol and triglycerides, along with the white blood cell count, were determined using the Advia 1800 Chemistry System and appertaining reagents (Siemens) according to the manufacturer’s specifications at the Veterinary Diagnostic Laboratory at the University of Copenhagen, Denmark.

### RNA extraction from blood samples

Total RNA was extracted from blood samples in PAXgene Blood RNA Tubes and treated with RNase-free DNase to remove genomic DNA (gDNA) using the PAXgene Blood miRNA Kit (PreAnalytix) according to the manufacturer’s specifications with the minor alteration of repeating the elution step by reusing the eluate. The process included on-column DNase treatment for elimination of gDNA. RNA quality was evaluated by the RNA integrity number (RIN) using the Agilent RNA 6000 Nano Kit on an Agilent 2100 Bioanalyzer (Agilent Technologies). The mean RIN was 8.2 (range 6.4–9.6).

### RNA extraction from liver and VAT

Approximately 30 mg of liver tissue or VAT was homogenized in 1 ml QIAzol Lysis Reagent (Qiagen) using M-tubes and a gentleMACS Dissociator (Miltenyi Biotec) and afterward thoroughly mixed with 200 μl chloroform (Emsure). From this point, total RNA was isolated according to the manufacturer’s instructions using the miRNeasy Mini Kit (Qiagen), including on-column DNase treatment for elimination of gDNA. The mean RIN for liver was 6.9 (range 6.0–7.9), while mean RIN for VAT was 8.1 (range 7.2–8.6).

### cDNA synthesis, preamplification and exonuclease treatment

Complementary DNA (cDNA) was synthesized in two replicates from each sample of isolated RNA from liver, VAT and whole blood using 500 ng total RNA per reaction. The QuantiTect Reverse Transcription Kit (Qiagen) was used for cDNA synthesis, using an optimized version of the manufacturer’s instructions. Alterations to the manufacturer’s instructions included using 1.5 µl gDNA Wipeout Buffer in the DNase treatment step and 0.75 μl Quantiscript Reverse Transcriptase, 0.75 μl RT Primer Mix and 3 μl Quantiscript RT Buffer in the cDNA synthesis step. cDNA samples were preamplified by combining 3 μl TaqMan PreAmp Master Mix (Applied Biosystems), 2.5 μl 200 mM mix of specific primers (Supplementary Tables 5 and 6) used in the subsequent quantitative PCR (qPCR), 2 μl low-EDTA TE buffer, and 2.5 μl diluted cDNA (1:10 in low-EDTA TE buffer (1×) (PanReac AppliChem)) per reaction. The mix was incubated at 95 °C for 10 min, followed by 19 cycles for blood samples and VAT and 18 cycles for liver samples of 95 °C for 10 s and 60 °C for 4 min. Preamplified cDNA was treated with 4 U Exonuclease I for 30 min at 37 °C, followed by 80 °C for 15 min, and diluted 1:10 in low-EDTA TE buffer before qPCR.

### qPCR

qPCR was carried out in Dynamic Array Integrated Fluid Circuit (IFC) chips on a BioMark real-time PCR platform (Standard Biotools). Twenty genes of interest in addition to four potential reference genes (Supplementary Table 5) were selected for quantification in all blood samples from days 0, 20, 41, 62, 83 and 98 on 192.24 Dynamic Array IFC chips for gene expression (Standard Biotools) on a BioMark real-time PCR platform (Standard Biotools) according to the manufacturer’s specifications. qPCR of liver and VAT samples was carried out on a 96.96 Dynamic Array IFC chip (Standard Biotools) on a BioMark real-time PCR platform according to the manufacturer’s specifications. A panel of 96 primer pairs representing 91 different genes was applied for quantification of gene expression in liver and VAT, including a selection of potential reference genes (Supplementary Table 6). The final primer concentration was 5 µM.

qPCR efficiency and dynamic range for each primer pair was determined on the basis of serial dilutions generated from individual pools made from cDNA from liver, VAT and blood samples, respectively. qPCR data were inspected in Fluidigm Real-Time PCR Analysis software 4.7.1 (Standard Biotools) and processed using GenEx7 Pro software (MultiD Analyses AB). Reverse-transcription qPCR of one VAT sample from the SD group was found to have been unsuccessful, leaving three VAT samples for gene expression analysis in this group. Qualification cycle (*C*_q_) values were corrected using PCR efficiencies (qPCR efficiencies between 90% and 110% were accepted) and normalized to the most stable reference genes identified using NormFinder^54^ and geNorm^55^. Actin beta (*ACTB*), glyceraldehyde-3-phosphate dehydrogenase (*GAPDH*) and tyrosine 3-monooxygenase (*YWHAZ*) and *ACTB*, *GAPDH*, ribosomal protein L13a (*RPL13A*), peptidylprolyl isomerase A (*PPIA*) and *YWHAZ* were used for data normalization in liver and VAT samples, respectively. cDNA replicates were averaged, and relative quantities were calculated by scaling data relative to the sample with the lowest expression for each assay.

### LPS quantitation by Limulus amebocyte lysate assay

LPS concentration, quantified by Limulus amebocyte lysate in serum samples on day 99, was determined in duplicates using Pierce Chromogenic Endotoxin Quant Kit (Thermo Fisher Scientific), following the manufacturer’s specifications. Serial dilutions of LPS in pretreated serum (diluted 1:100 in endotoxin-free water and heated at 70 °C for 15 min) from two HFFC-fed pigs and two SD-fed pigs were included in the assay to test for the presence of interfering substances in the serum.

### Histology

Liver tissue and VAT samples from four HFFC-fed and four SD-fed pigs were histologically examined by hematoxylin and eosin staining and Sudan III staining. Tissue samples were fixed in 10% neutral buffered formalin, embedded in paraffin wax, sectioned at 3–5 μm and stained with hematoxylin and eosin. Sudan III staining for lipids was performed on selected samples of the formalin-fixed tissues. The selected samples were transferred to 85% sucrose solution and frozen at −18 °C, then cryo-sectioned at 3–5 µm and stained by the Sudan III method.

The histopathological examination was performed blinded and systematically. A Leica DMRB microscope was used for reading, and images were obtained using a Leica MC120 HD camera and Leica Application Suite software, version 4.7.0 (Leica Microsystems).

### Data and statistical analysis

All data analysis was performed in a blinded manner. Data normality was assessed using the Shapiro–Wilk test. Mixed analysis of variance was used to test the effect of diet and time on leukocyte gene expression data. When Mauchly’s test indicated violation of sphericity, Greenhouse–Geisser corrections were applied. Post-hoc analyses used two-sided Welch’s *t*-tests with Benjamini–Hochberg correction (*α* = 0.05). Differences in BW, ABC, CRP, triglyceride, total cholesterol, LDL and HDL concentrations as well as neutrophil and monocyte cell counts were analyzed using two-sided Welch’s *t*-tests with Benjamini–Hochberg corrections. Differences in LPS concentrations between diet groups on day 99 were analyzed using two-sided Welch’s *t*-test. Transcriptional data from blood samples were log_2_-transformed before statistical analysis. Two-sided Welch’s *t*-test was used for post-hoc analyses, and the resulting *P* values were adjusted for all genes using the Benjamini–Hochberg correction (*α* = 0.05).

Correlation between CRP concentrations and BW, ABC, triglycerides, total cholesterol, LDL, HDL and LPS concentrations was determined using Spearman’s correlation coefficient, *r*_S_, with significance level *α* = 0.05. As sample sizes for transcriptional analysis of liver tissue and VAT were small (*n* = 4 for liver and VAT in the HFFC group; *n* = 4 for liver and *n* = 3 for VAT in the SD group), only descriptive statistics were applied. A criterion of ≥2-fold up- or downregulation, combined with nonoverlapping standard errors of the mean (s.e.m.), was used to identify genes that were differentially expressed between the HFFC and SD groups.

### Reporting summary

Further information on research design is available in the Nature Portfolio Reporting Summary linked to this article.

## Online content

Any methods, additional references, Nature Portfolio reporting summaries, source data, extended data, supplementary information, acknowledgements, peer review information; details of author contributions and competing interests; and statements of data and code availability are available at 10.1038/s41684-025-01588-3.

## Supplementary information

Supplementary Information Supplementary Tables 1–6.

Reporting Summary

## Data Availability

The data that support the findings of this study are available from the corresponding author upon request.
