# Supplementary material for: Meta-inflammation and endotoxemia in a highly translational porcine model of diet-induced obesity
Source: Lab Anim (NY). 2025 Aug 6;54(9):238–46. doi: 10.1038/s41684-025-01588-3 (PMC12404983; doi:10.1038/s41684-025-01588-3)
Supplement: Supplementary file 1 — Supplementary Tables 1–6. [file 41684_2025_1588_MOESM1_ESM.pdf]

lab animal

Supplementary information

https://doi.org/10.1038/s41684-025-01588-3

Meta-inflammation and endotoxemia in a highly translational porcine model of diet-induced obesity

In the format provided by the authors and unedited

Supplementary Table 1. Gene expression in VAT. Data is presented as mean relative expression ± SEM.

Gene	SD group - VAT<br>Relative expression level ± SEM	HFFC group - VAT<br>Relative expression level ± SEM
LTF	1 ± 0.3	9.9 ± 2.8
ABCA1	1 ± 0.2	4.7 ± 1.0
MPO	1 ± 0.3	3.3 ± 1.8
LEP (primer pair A)	1 ± 0.2	3.2 ± 0.8
LEP (primer pair B)	1 ± 0.3	3.0 ± 0.7
ABCG1	1 ± 0.2	2.9 ± 0.6
LPSBP	1 ± 0.5	2.6 ± 0.3
IL1A	1 ± 0.2	2.6 ± 0.3
IFNG	1 ± 0.2	2.3 ± 0.3
ORM1 (primer pair A)	1 ± 0.3	2.2 ± 0.5
ORM1 (primer pair B)	1 ± 0.6	2.1 ± 0.9
CD27	1 ± 0.1	2.1 ± 0.3
ALB	1 ± 0.1	2.0 ± 0.4
GHRL	1 ± 0.1	2.0 ± 0.4
IL18	1 ± 0.2	1.9 ± 0.1
FAS	1 ± 0.2	1.8 ± 0.3
SPP1	1 ± 0.4	1.7 ± 0.2
NOD2	1 ± 0.2	1.7 ± 0.4
MMP9	1 ± 0.7	1.7 ± 0.4
IL4	1 ± 0.2	1.7 ± 0.6
CCL4	1 ± 0.2	1.6 ± 0.1
KLB	1 ± 0.1	1.5 ± 0.1
VCAM	1 ± 0.1	1.5 ± 0.2
ADIPOQ	1 ± 0.1	1.5 ± 0.0
KLB	1 ± 0.1	1.5 ± 0.1
NOS2	1 ± 0.2	1.4 ± 0.2

TGFB1	1 ± 0.1	1.4 ± 0.1
APOA1	1 ± 0.1	1.4 ± 0.2
CD80	1 ± 0.3	1.4 ± 0.3
MMP8	1 ± 0.4	1.3 ± 0.4
IL10	1 ± 0.3	1.3 ± 0.1
IRS2	1 ± 0.1	1.3 ± 0.2
IRS1	1 ± 0.1	1.3 ± 0.2
SAA2	1 ± 0.2	1.3 ± 0.2
CD36	1 ± 0.1	1.3 ± 0.1
FABP4	1 ± 0.1	1.2 ± 0.3
FTO	1 ± 0.0	1.2 ± 0.1
CXCL8	1 ± 0.2	1.2 ± 0.3
NOD1	1 ± 0.2	1.2 ± 0.1
STAT3	1 ± 0.1	1.2 ± 0.1
CXCR4	1 ± 0.0	1.2 ± 0.2
CD68	1 ± 0.4	1.2 ± 0.1
RELA	1 ± 0.2	1.2 ± 0.1
TNF	1 ± 0.2	1.1 ± 0.5
PPARG2	1 ± 0.0	1.1 ± 0.1
IL6	1 ± 0.2	1.1 ± 0.2
CD163	1 ± 0.4	1.1 ± 0.2
LEPR	1 ± 0.9	1.1 ± 0.8
CRP	1 ± 0.2	1.1 ± 0.1
TBP	1 ± 0.1	1.1 ± 0.0
CXCL2	1 ± 0.0	1.1 ± 0.5
IL1RAP	1 ± 0.2	1.0 ± 0.0
C1QB	1 ± 0.2	1.0 ± 0.1
ADIPOR1	1 ± 0.1	1.0 ± 0.1
NFKBIA	1 ± 0.1	1.0 ± 0.2
MTOR	1 ± 0.1	1.0 ± 0.1
RARRES2	1 ± 0.2	1.0 ± 0.2

IRF5	1 ± 0.3	1.0 ± 0.1
IRF1	1 ± 0.2	1.0 ± 0.1
NFKB1	1 ± 0.0	1.0 ± 0.1
MMP2	1 ± 0.2	1.0 ± 0.1
CCL3	1 ± 0.3	1.0 ± 0.2
C3	1 ± 0.4	0.9 ± 0.3
FGG	1 ± 0.3	0.9 ± 0.2
SAA3	1 ± 0.3	0.9 ± 0.4
TLR4	1 ± 0.2	0.9 ± 0.1
CCL2	1 ± 0.3	0.9 ± 0.1
C5	1 ± 0.1	0.8 ± 0.0
TGFB2	1 ± 0.0	0.8 ± 0.1
HP	1 ± 0.0	0.8 ± 0.3
CHI3L1	1 ± 0.3	0.8 ± 0.2
ICAM1	1 ± 0.2	0.8 ± 0.1
CD86	1 ± 0.4	0.8 ± 0.1
IRF4	1 ± 0.4	0.8 ± 0.1
S100A8	1 ± 0.2	0.7 ± 0.1
S100A12	1 ± 0.2	0.7 ± 0.1
TF	1 ± 0.2	0.7 ± 0.5
NLRP3	1 ± 0.2	0.7 ± 0.1
IL1B	1 ± 0.2	0.7 ± 0.1
IL1B	1 ± 0.3	0.7 ± 0.1
SERPINE1	1 ± 0.3	0.6 ± 0.0
CASP1	1 ± 0.4	0.6 ± 0.1
IL1RN	1 ± 0.4	0.5 ± 0.1
FASN	1 ± 0.4	0.5 ± 0.1
CXCL14	1 ± 0.2	0.5 ± 0.0
SCD1	1 ± 0.3	0.4 ± 0.1
IRF7	1 ± 0.1	0.4 ± 0.0
CXCL10	1 ± 0.7	0.4 ± 0.1

TTR	1 ± 0.9	0.2 ± 0.1

HFFC, high fat, fructose, and cholesterol diet. SD, standard diet. SEM, standard error of the mean.

Supplementary Table 2. Gene expression in circulating leukocytes. Data is presented as mean relative expression ± SEM.

Gene	Day 0		Day 20		Day 41		Day 62		Day 83		Day 98		Overall p-value
	SD group leukocytes Relative expression level ± SEM	HFFC group - leukocytes Relative expression level ± SEM	SD group leukocytes Relative expression level ± SEM	HFFC group - leukocytes Relative expression level ± SEM	SD group leukocytes Relative expression level ± SEM	HFFC group - leukocytes Relative expression level ± SEM	SD group leukocytes Relative expression level ± SEM	HFFC group - leukocytes Relative expression level ± SEM	SD group leukocytes Relative expression level ± SEM	HFFC group - leukocytes Relative expression level ± SEM	SD group leukocytes Relative expression level ± SEM	HFFC group - leukocytes Relative expression level ± SEM	
ABCA1	1.0 ± 0.1	0.8 ± 0.1	0.8 ± 0.1	6.7 ± 0.6	0.9 ± 0.1	6.4 ± 0.4	1.2 ± 0.2	9.9 ± 0.8	1.6 ± 0.6	11.9 ± 1.1	1.4 ± 0.1	8.4 ± 0.6	4,04E-05
ADIPOR1	1.0 ± 0.2	1.6 ± 0.2	0.8 ± 0.1	1.0 ± 0.1	0.6 ± 0.1	1.4 ± 0.1	0.6 ± 0.1	2.9 ± 0.2	1.1 ± 0.5	1.4 ± 0.1	1.6 ± 0.2	1.8 ± 0.1	3,64E-03
CD14	1.0 ± 0.2	0.8 ± 0.1	1.2 ± 0.1	1.8 ± 0.3	1.3 ± 0.1	1.5 ± 0.2	1.3 ± 0.1	1.3 ± 0.1	1.4 ± 0.2	2.1 ± 0.3	2.1 ± 0.3	2.0 ± 0.3	2,84E-06
CD163	1.0 ± 0.1	0.9 ± 0.2	0.8 ± 0.1	2.2 ± 0.3	0.9 ± 0.1	2.1 ± 0.2	0.9 ± 0.1	2.5 ± 0.3	1.0 ± 0.1	3.9 ± 0.4	1.8 ± 0.2	3.7 ± 0.5	8,90E-06
CXCL8	1.0 ± 0.3	1.3 ± 0.5	1.2 ± 0.2	0.9 ± 0.2	0.9 ± 0.2	0.9 ± 0.1	0.9 ± 0.3	1.5 ± 0.3	1.3 ± 0.8	0.8 ± 0.2	0.9 ± 0.1	0.8 ± 0.1	1,58E-02
HP	1.0 ± 0.1	1.0 ± 0.2	1.0 ± 0.1	1.3 ± 0.2	1.2 ± 0.1	1.3 ± 0.1	1.0 ± 0.1	1.6 ± 0.1	0.9 ± 0.1	2.0 ± 0.3	1.5 ± 0.1	2.9 ± 0.6	4,84E-04
IL18	1.0 ± 0.2	1.4 ± 0.3	1.3 ± 0.2	1.6 ± 0.3	1.1 ± 0.2	1.6 ± 0.2	1.1 ± 0.1	1.5 ± 0.3	1.4 ± 0.2	2.5 ± 0.5	2.3 ± 0.4	2.7 ± 0.6	6,75E-01
IL1A	1.0 ± 0.1	1.1 ± 0.2	0.9 ± 0.1	1.2 ± 0.3	0.7 ± 0.1	0.6 ± 0.1	0.7 ± 0.0	0.8 ± 0.1	2.8 ± 1.9	1.1 ± 0.2	1.2 ± 0.2	1.1 ± 0.1	6,38E-03
IL1RAP	1.0 ± 0.1	1.1 ± 0.2	0.8 ± 0.1	1.2 ± 0.2	0.7 ± 0.0	0.8 ± 0.1	0.7 ± 0.0	0.9 ± 0.1	0.9 ± 0.3	0.9 ± 0.1	1.1 ± 0.1	1.2 ± 0.2	4,10E-01
IL4	1.0 ± 0.2	0.8 ± 0.2	1.8 ± 0.3	2.2 ± 0.4	2.2 ± 0.2	5.8 ± 0.9	2.0 ± 0.3	6.0 ± 1.3	1.4 ± 0.2	6.2 ± 1.2	1.9 ± 0.3	5.1 ± 0.6	3,72E-05
IL6	1.0 ± 0.2	0.7 ± 0.2	1.1 ± 0.3	1.0 ± 0.2	0.7 ± 0.2	1.3 ± 0.3	0.9 ± 0.2	1.2 ± 0.2	0.4 ± 0.1	1.7 ± 0.5	1.1 ± 0.3	1.2 ± 0.3	1,82E-01
IRF1	1.0 ± 0.1	1.1 ± 0.1	0.9 ± 0.1	1.4 ± 0.1	1.0 ± 0.1	1.3 ± 0.1	1.1 ± 0.0	1.3 ± 0.1	1.2 ± 0.4	1.0 ± 0.0	1.0 ± 0.1	1.1 ± 0.1	1,86E-01

LTF	1.0 ± 0.3	0.9 ± 0.2	0.7 ± 0.1	2.4 ± 0.4	0.5 ± 0.1	1.3 ± 0.2	0.4 ± 0.1	2.1 ± 0.3	0.4 ± 0.1	3.7 ± 0.5	0.9 ± 0.2	3.6 ± 0.7	3,71E-03
LY96	1.0 ± 0.1	0.9 ± 0.1	0.9 ± 0.1	1.2 ± 0.2	0.6 ± 0.1	0.9 ± 0.1	0.6 ± 0.0	0.9 ± 0.1	1.5 ± 0.8	1.2 ± 0.1	0.9 ± 0.1	1.0 ± 0.1	3,52E-02
MMP9	1.0 ± 0.2	0.8 ± 0.3	0.9 ± 0.2	0.7 ± 0.3	3.9 ± 1.0	0.6 ± 0.1	1.8 ± 0.3	0.6 ± 0.1	1.9 ± 0.3	0.4 ± 0.1	1.8 ± 0.4	0.5 ± 0.2	1,34E-01
NFKB1	1.0 ± 0.0	1.0 ± 0.1	1.0 ± 0.1	1.3 ± 0.1	1.0 ± 0.0	1.5 ± 0.1	1.1 ± 0.0	1.4 ± 0.1	1.3 ± 0.1	1.5 ± 0.1	1.2 ± 0.1	1.3 ± 0.1	4,43E-03
RETN	1.0 ± 0.2	1.1 ± 0.3	0.9 ± 0.1	1.6 ± 0.2	1.0 ± 0.1	1.3 ± 0.2	0.7 ± 0.1	1.1 ± 0.1	1.0 ± 0.2	1.6 ± 0.2	1.5 ± 0.2	2.3 ± 0.6	8,91E-05
S100A8	1.0 ± 0.2	0.8 ± 0.3	0.9 ± 0.1	2.0 ± 0.3	0.9 ± 0.1	1.5 ± 0.2	0.6 ± 0.1	1.5 ± 0.2	1.1 ± 0.3	3.4 ± 0.3	1.3 ± 0.2	2.8 ± 0.4	7,09E-06
S100A12	1.0 ± 0.2	1.0 ± 0.3	0.8 ± 0.1	1.7 ± 0.2	0.8 ± 0.1	1.2 ± 0.2	0.7 ± 0.1	1.3 ± 0.1	0.7 ± 0.1	2.5 ± 0.3	1.4 ± 0.2	2.6 ± 0.4	1,41E-04
TNF	1.0 ± 0.1	0.9 ± 0.1	0.9 ± 0.0	1.0 ± 0.0	0.9 ± 0.0	0.9 ± 0.1	1.2 ± 0.0	1.2 ± 0.1	2.8 ± 1.6	1.2 ± 0.1	1.2 ± 0.1	1.2 ± 0.1	1,76E-03

HFFC, high fat, fructose, and cholesterol diet. SD, standard diet. SEM, standard error of the mean.

Supplementary Table 3. Gene expression in the liver. Data is presented as mean relative expression ± SEM.

Gene	SD group - VAT<br>Relative expression level ± SEM	HFFC group - VAT<br>Relative expression level ± SEM
CXCL14	1 ± 0.1	113.6 ± 25.8
PPARG2	1 ± 0.3	53.7 ± 11.5
ABCG1	1 ± 0.2	35.8 ± 4.0
SAA3	1 ± 0.4	27.8 ± 13.4
MMP9	1 ± 0.5	20.2 ± 11.3
SPP1	1 ± 0.2	13.0 ± 2.3
CD36	1 ± 0.2	7.7 ± 2.2
CD68	1 ± 0.1	7.3 ± 1.7
FAS	1 ± 0.2	6.9 ± 1.8
CXCL8	1 ± 0.2	6.6 ± 1.9
S100A8	1 ± 0.3	6.3 ± 0.8
CXCL10	1 ± 0.4	6.2 ± 2.4
CXCR4	1 ± 0.2	6.0 ± 1.8
CD86	1 ± 0.1	5.6 ± 0.5
LTF	1 ± 0.1	4.0 ± 1.1
CCL3	1 ± 0.2	3.8 ± 0.4
S100A12	1 ± 0.2	3.8 ± 0.5
MPO	1 ± 0.5	3.7 ± 0.5
C1QB	1 ± 0.1	3.6 ± 0.6
NOD1	1 ± 0.2	3.6 ± 0.4
TLR4	1 ± 0.1	3.5 ± 0.8
NLRP3	1 ± 0.1	3.4 ± 0.7
CD80	1 ± 0.2	3.4 ± 0.8
NOS2	1 ± 0.3	3.2 ± 0.7

IFNG	1 ± 0.3	3.0 ± 0.6
IRF5	1 ± 0.1	3.0 ± 0.5
ABCA1	1 ± 0.1	2.8 ± 0.3
NOD2	1 ± 0.2	2.7 ± 1.3
CXCL2	1 ± 0.7	2.5 ± 1.1
TNF	1 ± 0.1	2.5 ± 0.5
SCD1	1 ± 0.1	2.4 ± 0.4
CD27	1 ± 0.1	2.2 ± 0.3
CCL4	1 ± 0.1	2.2 ± 0.4
IL1A	1 ± 0.1	2.2 ± 1.0
ICAM1	1 ± 0.2	2.0 ± 0.5
TGFB1	1 ± 0.1	1.9 ± 0.2
IL10	1 ± 0.3	1.9 ± 0.3
IRF4	1 ± 0.2	1.9 ± 0.7
IL6	1 ± 0.3	1.8 ± 0.5
MMP2	1 ± 0.1	1.7 ± 0.4
CD163	1 ± 0.2	1.7 ± 0.6
MMP8	1 ± 0.2	1.6 ± 0.4
IL4	1 ± 0.2	1.6 ± 0.5
IRF7	1 ± 0.3	1.6 ± 0.2
LEPR	1 ± 0.7	1.6 ± 0.8
FABP4	1 ± 0.3	1.5 ± 0.4
RELA	1 ± 0.1	1.5 ± 0.4
SERPINE1	1 ± 0.3	1.4 ± 0.2
VCAM	1 ± 0.2	1.4 ± 0.2
IL18	1 ± 0.2	1.4 ± 0.3
CCL2	1 ± 0.3	1.4 ± 0.3
CHI3L1	1 ± 0.3	1.3 ± 0.1
IRF1	1 ± 0.3	1.3 ± 0.1

MTOR	1 ± 0.1	1.2 ± 0.0
TGFB2	1 ± 0.0	1.2 ± 0.2
IL1B	1 ± 0.3	1.1 ± 0.3
SAA2	1 ± 0.5	1.1 ± 0.4
IRS1	1 ± 0.0	1.1 ± 0.2
TBP	1 ± 0.0	1.0 ± 0.2
FTO	1 ± 0.1	1.0 ± 0.1
NFKB1	1 ± 0.1	1.0 ± 0.1
C3	1 ± 0.2	1.0 ± 0.2
IL1RN	1 ± 0.1	0.9 ± 0.1
APOA1	1 ± 0.1	0.9 ± 0.2
CASP1	1 ± 0.2	0.9 ± 0.2
IL1B	1 ± 0.2	0.9 ± 0.2
CRP (primer pair A)	1 ± 0.4	0.9 ± 0.3
ADIPOR1	1 ± 0.1	0.8 ± 0.1
TTR	1 ± 0.2	0.8 ± 0.2
RARRES2	1 ± 0.1	0.7 ± 0.2
ALB	1 ± 0.2	0.7 ± 0.2
STAT3	1 ± 0.1	0.7 ± 0.1
NFKBIA	1 ± 0.6	0.6 ± 0.1
IRS2	1 ± 0.1	0.6 ± 0.1
TF	1 ± 0.2	0.5 ± 0.2
IL1RAP	1 ± 0.1	0.5 ± 0.1
CRP (primer pair B)	1 ± 0.4	0.5 ± 0.1
ORM1 (primer pair A)	1 ± 0.1	0.5 ± 0.0
LPSBP	1 ± 0.2	0.5 ± 0.2
HP	1 ± 0.2	0.5 ± 0.2
FGG	1 ± 0.1	0.5 ± 0.1
ORM1 (primer pair B)	1 ± 0.2	0.5 ± 0.0
MBL2	1 ± 0.1	0.5 ± 0.2
FASN	1 ± 0.2	0.3 ± 0.1

KLB (primer pair A)	1 ± 0.3	0.01 ± 0.0
KLB (primer pair B)	1 ± 0.3	0.01 ± 0.0

HFFC, high fat, fructose, and cholesterol diet. SD, standard diet. SEM, standard error of the mean.

Supplementary Table 4. Diet compositions. Percentages are of total energy content.

Diet components	Standard diet (SD)	High fat, fructose, and cholesterol diet (5B4L) (HFFC)
Carbohydrates (%)	74.6	40.8
Protein (%)	18.6	16.2
Fat (%)	6.8	43.0
Fructose (%)	< 5.5	17.8
Cholesterol (ppm)	~ 0	20,045
Choline (ppm)	784	668

Supplementary Table 5. qPCR primer sequences for genes studied in total leukocytes.

Gene of interest	Primers	Efficiency	Transcripts covered	Intron spanning?	Amplicon length
ABCA1	F-CCTTCTGCTGTATGGGTGGT<br>R-GTGAGGACCACGTAGGCTGT	108 %	ENSSSCT00000005966.5	Intron 38-39	90 bp
ACTB	F-CTACGTCGCCCTGGACTTC<br>R-GCAGCTCGTAGCTCTTCTCC	101 %	ENSSSCT00000042531.2<br>ENSSSCT00000049015.3	-	77 bp
ADIPOR1	F- CCATGGAGAAGATGGAGGAG<br>R- GTCGTTGTCCTTCAGCCAGT	103 %	ENSSSCT00000081070.2<br>ENSSSCT00000069455.2<br>ENSSSCT00000069245.2<br>ENSSSCT00000011961.5	Intron 3-4<br>Intron 1-2<br>Intron 3-4<br>Intron 2-3	98 bp
B2M	F-TGAAGCACGTGACTCTCGAT<br>R-CTCTGTGATGCCGGTTAGTG	-	ENSSSCT00000104592.2<br>ENSSSCT00000107013.1<br>ENSSSCT00000107012.1<br>ENSSSCT00000084189.3<br>ENSSSCT00000107014.1<br>ENSSSCT00000087633.1	Intron 2-3<br>Intron 2-3<br>Intron 2-3<br>Intron 2-3<br>Intron 2-3<br>Intron 2-3	65 bp
CD14	F-AAGCTCACCGTGCTTGATCT<br>R-CCTTCCAGGGTCAGGTCAT	98 %	ENSSSCT00000015699.6	-	92 bp
CD163	F-CACATGTGCCAACAAAATAAGAC<br>R-CACCACCTGAGCATCTTCAA	104 %	ENSSSCT00000044319.3<br>ENSSSCT00000037917.3<br>ENSSSCT00000049020.2<br>ENSSSCT00000044540.2	Intron 12-13<br>Intron 11-12<br>Intron 8-9<br>Intron 11-12	130 bp
HP	F-ACAGATGCCACAGATGACAGC<br>R-CGTGCGCAGTTTGTAGTAGG	109 %	ENSSSCT00045037868.1<br>ENSSSCT00045037583.1<br>ENSSSCT00045037657.1<br>ENSSSCT00045037821.1<br>ENSSSCT00045037735.1	Intron 2-3<br>Intron 3-4<br>Intron 2-3<br>Intron 2-3<br>Intron 2-3	105 bp
CXCL8	F-TTGCCAGAGAAATCACAGGA<br>R-TGCATGGGACACTGGAAATA	106 %	ENSSSCT00000009807.5<br>ENSSSCT00000068386.1	-	78 bp
IL18	F-CTGCTGAACCGGAAGACAAT<br>R-TCCGATTCCAGGTCTTCATC	102 %	ENSSSCT00000083545.2<br>ENSSSCT00000047954.3	Intron 2-3<br>Intron 1-2	88 bp<br>100 bp
IL1A	F-TGTGCTAAATAACCTGGATGAGG<br>R-GGTTCGTCTTCGTTTTGAGC	101 %	ENSSSCT00000008863.4	Intron 5-6	135 bp
IL1RAP	F-GCATCACCTCCCCAAATCTA	103 %	ENSSSCT00000046997.3	Intron 7-8	70 bp

	R-GTAGCTCCTCTCCCGGTTCT				
IL4	F-GCAAACATGACCTGTTCTGTG<br>R-GCTTCAACACTTTGAGTATTTCTCC	104 %	ENSSSCT00000015605.3	Intron 3-4	105 bp
IL6	F-CCTCTCCGGACAAAACTGAA<br>R-TCTGCCAGTACCTCCTTGCT	96 %	ENSSSCT00000025647.4<br>ENSSSCT00000023544.4	Intron 2-3	118 bp
IRF1	F-TGAAGCTGCAACAGATGAGG<br>R-CTTCCCATCCACGTTTGTCT	105 %	ENSSSCT00000015600.6<br>ENSSSCT00000098417.1	Intron 7-8/8-9<br>Intron 7-8/8-9	100 bp
LTF	F-GGAAAAGACTGCCCAGACAA<br>R-ACACTCCGTGTTGTCGTTGA	100 %	ENSSSCT00000030564.3<br>ENSSSCT00000101357.1<br>ENSSSCT00000089932.1	-	78 bp
LY96	F-CAGTAAAGGTTGAGCCCTGTG<br>R-TTTGCGCATTGGTAAAGTCA	96 %	ENSSSCT00000006769.4	Intron 2-3	140 bp
MMP9	F-ACACACGACATCTTCCAGTACC<br>R-GTCCACCTGATTCACCTCGT	108 %	ENSSSCT00000049521.3	Intron 12-13	96 bp
NFKB1	F-CCCTGTGAAGACCACCTCTC<br>R-ATCCCGGAGCTCGTCTATTT	101 %	ENSSSCT00000038683.3	Intron 23-24	82 bp
PPIA	F-CAAGACTGAGTGGTTGGATGG<br>R-TGTCCACAGTCAGCAATGGT	107 %	ENSSSCT00000018219.5<br>ENSSSCT00000082244.2<br>ENSSSCT00000085168.2	Intron 4-5<br>Intron 5-6<br>Intron 4-5	138 bp
RETN	F-TTAGGAACATTGGCCTGGAA<br>R-CAGTGACAGCAAAGCCTGAG	101 %	ENSSSCT00000033014.3	Intron 3-4	81 bp
S100A12	F-TGAAGCAGCTGATCACCAAG<br>R-GTCTTGATTGGCATCCAGGT	110 %	ENSSSCT00000066217.3	Intron 2-3	101 bp
S100A8	F-ATGCTGACGGATCTGGAGAG<br>R-GGCGTGGTAATTCCCTTTCT	101 %	ENSSSCT00000036517.4	-	84 bp
TNF	F-CCCCCAGAAGGAAGAGTTTC<br>R-CGGGCTTATCTGAGGTTTGA	98 %	ENSSSCT00000104535.2	Intron 1-2/2-3	92 bp
YWHAZ	F-GCTGCTGGTGATGATAAGAAGG<br>R-AGTTAAGGGCCAGACCCAAT	105 %	ENSSSCT00000006651.4<br>ENSSSCT00000106709.1<br>ENSSSCT00000037127.3<br>ENSSSCT00000046817.3<br>ENSSSCT00000045155.2<br>ENSSSCT00000099418.1	Intron 3-4<br>Intron 5-6<br>Intron 5-6<br>Intron 5-6<br>Intron 2-3<br>Intron 3-4	124 bp

Supplementary Table 6. qPCR primer sequences for genes studied in VAT and liver.

Gene of interest	Primers	Efficiency VAT	Efficiency liver	Transcripts covered	Intron spanning?	Amplicon length
ABCA1	F-CCTTCTGCTGTATGGGTGGT<br>R-GTGAGGACCACGTAGGCTGT	108 %	105 %	ENSSSCT00000005966.5	Intron 38-39	90 bp
ABCG1	F-CCTGCAGTGGATGTCCTACA<br>R-AGGCCGTAGATGGAGAGGAT	103 %	104 %	ENSSSCT00000055448.3<br>ENSSSCT00000028391.3	Intron 14-15<br>Intron 15-16	72 bp
ACTB	F-CTACGTCGCCCTGGACTTC<br>R-GCAGCTCGTAGCTCTTCTCC	119 %	112 %	ENSSSCT00000042531.2<br>ENSSSCT00000049015.3	-	77 bp
ADIPOQ	F-AACATGCCCATTCGCTTTAC<br>R-AGACCGTGATGTGGAAGGAG	104 %	-	ENSSSCT00000038265.3<br>ENSSSCT00000047495.3	-	121 bp
ADIPOR1	F-CCATGGAGAAGATGGAGGAG<br>R-GTCGTTGTCCTTCAGCCAGT	115 %	118 %	ENSSSCT00000069245.2<br>ENSSSCT00000069455.2<br>ENSSSCT00000081070.2<br>ENSSSCT00000011961.5	Intron 3-4<br>Intron 2-3<br>Intron 3-4<br>Intron 2-3	98 bp
ALB	F-CAGAAGTTTTGGGGAAAATACCT<br>R-TAAAGGAGTTCTGGGGCGTA	99 %	116 %	ENSSSCT00000057761.3<br>ENSSSCT00000099055.1<br>ENSSSCT00000064748.3<br>ENSSSCT00000009802.5<br>ENSSSCT00000050096.3<br>ENSSSCT00000093326.1	Intron 7-8<br>Intron 4-5<br>Intron 4-5<br>Intron 4-5<br>Intron 4-5<br>Intron 4-5	74 bp
APOA1	F-GTTCTGGGACAACCTGGAAA<br>R-GCTGCACCTTCTTCTTCACC	105 %	116 %	ENSSSCT00000053994.2<br>ENSSSCT00000034599.4	-	86 bp
C1QB	F-GGGGATCAAGGGAGAGAAAG<br>R-CTCCCTTCTCTCCAACCTCA	110 %	101 %	ENSSSCT00000084461.2<br>ENSSSCT00000064269.2	Intron 2-3<br>Intron 2-3	276 bp<br>68 bp
C3	F-ATCAAATCAGGCTCCGATGA<br>R-GGGCTTCTCTGCATTTGATG	109 %	107 %	ENSSSCT00000101941.1<br>ENSSSCT00000087825.2<br>ENSSSCT00000073360.2<br>ENSSSCT00000103277.1<br>ENSSSCT00000023681.4<br>ENSSSCT00000101892.1<br>ENSSSCT00000087663.2<br>ENSSSCT00000078048.2	Intron 40-41<br>Intron 40-41<br>Intron 41-42<br>Intron 42-43<br>Intron 41-42<br>Intron 41-42<br>Intron 38-39<br>Intron 39-40	76 bp
C5	F-AAGCTGGAGAAGCCGTTGC	98 %	110 %	ENSSSCT00000042810.2	Intron 42-43	82 bp

	R-TTTTCGAGGTTAGCGTTCGT			ENSSSCT00000064155.2<br>ENSSSCT00000044735.3<br>ENSSSCT00000006062.5	Intron 41-42<br>Intron 39-40<br>Intron 38-39	
CASP1	F-GAAGGACAAACCCAAGGTGA<br>R-TGGGCTTTCTTAATGGCATC	106 %	111 %	ENSSSCT00000081027.2<br>ENSSSCT00000079620.2<br>ENSSSCT00000076386.1<br>ENSSSCT00000070445.1<br>ENSSSCT00000027757.3<br>ENSSSCT00000061467.3	Intron 6-7<br>Intron 7-8<br>Intron 5-6<br>Intron 5-6<br>Intron 7-8<br>Intron 7-8	147 bp
CCL2	F-CTTCTGCACCCAGGTCCTT<br>R-CGCTGCATCGAGATCTTCTT	107 %	112 %	ENSSSCT00000019290.5	Intron 1-2	93 bp
CCL3	F-CTCTGCAGCCAGGTCTTCTC<br>R-CTACGAATTTGCGAGGAAGC	105 %	104 %	ENSSSCT00000019266.6	Intron 1-2	97 bp
CCL4	F-CCGTGGTATTCCAGACCAAA<br>R-ACTCCTGGACCCAGTCATCA	107 %	107 %	ENSSSCT00000102499.1<br>ENSSSCT00000065828.3	Intron 2-3<br>Intron 3-4	69 bp
CD163	F-CACATGTGCCAACAAAATAAGAC<br>R-CACCACCTGAGCATCTTCAA	108 %	109 %	ENSSSCT00000044319.3<br>ENSSSCT00000037917.3<br>ENSSSCT00000049020.2<br>ENSSSCT00000044540.2	Intron 12-13<br>Intron 11-12<br>Intron 8-9<br>Intron 11-12	130 bp
CD27	F-GCAGGTTCCTGTCCCAACTA<br>R-CTCCAGAGAGGACCACAAGG	103 %	95 %	ENSSSCT00000102965.1	Intron 4-5	89 bp
CD36	F-CACTGTTCTCAATCTGGCTGTG<br>R-CCGGATAGCCCCACAATAG	107%	103 %	ENSSSCT00000103672.1<br>ENSSSCT00000101008.1<br>ENSSSCT00000104709.1<br>ENSSSCT00000094054.1<br>ENSSSCT00000034552.4<br>ENSSSCT00000103448.1<br>ENSSSCT00000016780.5<br>ENSSSCT00000078461.2<br>ENSSSCT00000105268.1	Intron 6-7<br>Intron 6-7<br>Intron 6-7<br>Intron 6-7<br>Intron 5-6<br>Intron 3-4<br>Intron 5-6<br>Intron 5-6<br>Intron 3-4	146 bp
CD68	F-CATGGCTGTGGAGTACAACG<br>R-TGGAGATCTCGAAGGGATGA	104 %	104 %	ENSSSCT00000019540.5	Intron 4-5	84 bp
CD80	F-CGCACCTTCACTGATGTCAC<br>R-CACAGGTGTAGGTGCCATTG	95 %	103 %	ENSSSCT00000013021.6<br>ENSSSCT00000034815.3<br>ENSSSCT00000102192.1	-	82 bp
CD86	F-CATCGTCTGTGTCCTGCAAC	108 %	114 %	ENSSSCT00000034076.4	Intron 4-5	82 bp

	R-CACAGGTGGCTTTGCATCTA			ENSSSCT00000080729.1	Intron 4-5	
CHI3L1	F-GGAGAGCGTCAAAAACAAGG<br>R-GTCCAGGGTCCACACCATAG	107 %	80 %	ENSSSCT00000048761.3<br>ENSSSCT00000092050.1<br>ENSSSCT00000016859.5	Intron 9-10<br>Intron 9-10<br>Intron 9-10	73 bp
CRP (primer pair A)	F-GGTGGGAGACATTGGAGATG<br>R-GAAGGTCCCACCAGCATAGA	100 %	111 %	ENSSSCT00000072472.2<br>ENSSSCT00000007016.5<br>ENSSSCT00000054270.3<br>ENSSSCT00000090287.2	-	85 bp
CRP (primer pair B)	F-CTTTTGCCCAGACAGACATGAT<br>R-GAGTGGTTTGGTGAGCCTTG	107 %	107 %	ENSSSCT00000007016.5	Intron 1-2	98 bp
CXCL10	F-CCCACATGTTGAGATCATTGC<br>R-GCTTCTCTCTGTGTTCGAGGA	105 %	108 %	ENSSSCT00000062500.2	Intron 2-3/3-4	141 bp
CXCL14	F-GTACCGAGGTCAGGAGCACT<br>R-TAGACCCTGCGCTTCTCATT	104 %	115 %	ENSSSCT00000015634.6	Intron 3-4	96 bp
CXCL2	F-AGGAATTCACCTCAAGAACATCC<br>R-GCTGAGGGGTTGAGACAAAC	118 %	113 %	ENSSSCT00000040395.3	Intron 2-3	120 bp
CXCL8	F-TTGCCAGAGAAATCACAGGA<br>R-TGCATGGGACACTGGAAATA	98 %	106 %	ENSSSCT00000009807.5<br>ENSSSCT00000068386.1	-	78 bp
CXCR4	F-ACGGGTTCCGTATATTCACTTC<br>R-GGAAACAGGGTTCCTTTATGG	102 %	104 %	ENSSSCT00000064385.3	Intron 1-2	87 bp
FABP4	F-GCAGAAGTGGGATGGAAAGAC<br>R-ATTCTGGTAGCCGTGACACC	107 %	94 %	ENSSSCT00000064366.3	Intron 3-4	99 bp
FAS	F-CACTGTAACCCTTGCACCAC<br>R-TGGAACACTTCTCTGCATTTGG	105 %	100 %	ENSSSCT00000011433.5<br>ENSSSCT00000073478.1<br>ENSSSCT00000086188.1	Intron 5-6/6-7<br>Intron 4-5/5-6<br>Intron 4-5/5-6	86 bp
FASN	F-CCTGGCCTCCTACTACATCG<br>R-CAGGCCCCGTAAGAGTAGC	114 %	113 %	ENSSSCT00000026033.4<br>ENSSSCT00000029960.4	-	84 bp
FGG	F-CAAAACCAGATAGGATACAAAGTGC<br>R-CGAATCGTTGAGTCGTGTGT	109 %	108 %	ENSSSCT00000069628.2<br>ENSSSCT00000026223.4<br>ENSSSCT00000009856.4	Intron 3-4<br>Intron 3-4<br>Intron 3-4	103 bp
FTO	F-CTCAGCTGGAAGAGCTTTGG<br>R-TCACTGCTCTGTTCCACAGG	112 %	112 %	ENSSSCT00000038045.2<br>ENSSSCT00000080519.1<br>ENSSSCT00000051015.2<br>ENSSSCT00000062853.3	Intron 7-8<br>Intron 7-8<br>Intron 8-9<br>Intron 8-9	97 bp
GAPDH	F-ACCCAGAAGACTGTGGATGG<br>R-AAGCAGGGATGATGTTCTGG	109 %	107 %	ENSSSCT00000000756.4<br>ENSSSCT00000043459.3	-	79 bp

GHRL	F-CAAGTTGTCAGGGGCTCAGT<br>R-CCAGAGGATGTCCTGGAGAA	100 %	-	ENSSSCT00000012660.5<br>ENSSSCT00000075069.2<br>ENSSSCT00000068335.2	-	70 bp
HP	F-ACAGATGCCACAGATGACAGC<br>R-CGTGCGCAGTTTGTAGTAGG	117 %	116 %	ENSSSCT00045037868.1<br>ENSSSCT00045037583.1<br>ENSSSCT00045037657.1<br>ENSSSCT00045037821.1<br>ENSSSCT00045037735.1	Intron 2-3<br>Intron 3-4<br>Intron 2-3<br>Intron 2-3<br>Intron 2-3	105 bp
ICAM1	F-AAGCTTCTCCTGCTCTGCTG<br>R-GGGGTCCATACAGGACACTG	107 %	117 %	ENSSSCT00000032707.3<br>ENSSSCT00000072788.1	Intron 5-6<br>Intron 5-6	89 bp
IFNG	F-CCATTCAAAGGAGCATGGAT<br>R-TTCAGTTTCCCAGAGCTACCA	102 %	109 %	ENSSSCT00000055560.3	-	76 bp
IL10	F-TACAACAGGGGCTTGCTCTT<br>R-GCCAGGAAGATCAGGCAATA	107 %	97 %	ENSSSCT00000017049.6	-	110 bp
IL18	F-CTGCTGAACCGGAAGACAAT<br>R-TCCGATTCCAGGTCTTCATC	101 %	109 %	ENSSSCT00000083545.2<br>ENSSSCT00000047954.3	Intron 2-3<br>Intron 1-2	88 bp<br>100 bp
IL1A	F-TGTGCTAAATAACCTGGATGAGG<br>R-GGTTCGTCTTCGTTTTGAGC	102 %	107 %	ENSSSCT00000008863.4	Intron 5-6	135 bp
IL1B (primer pair A)	F-CCAAAGAGGGACATGGAGAA<br>R-GGGCTTTTGTTCTGCTTGAG	111 %	100 %	ENSSSCT00035048529.1<br>ENSSSCT00035048522.1	-	123 bp
IL1B (primer pair B)	F-TCTCTCACCCCTTCTCCTCA<br>R-GACCCTAGTGTGCCATGGTT	109 %	104 %	ENSSSCT00035048529.1<br>ENSSSCT00035048522.1	-	60 bp
IL1RAP	F-GCATCACCTCCCCAAATCTA<br>R-GTAGCTCCTCTCCCGGTTCT	115 %	103 %	ENSSSCT00000046997.3	Intron 7-8	70 bp
IL1RN	F-TGCCTGTCCTGTGTCAAGTC<br>R-GTCCTGCTCGCTGTTCTTTC	101 %	105 %	ENSSSCT00115016706.1	Intron 4-5	90 bp
IL4	F-GCAAACATGACCTGTTCTGTG<br>R-GCTTCAACACTTTGAGTATTTCTCC	104 %	107 %	ENSSSCT00000015605.3	Intron 3-4	105 bp
IL6	F-CCTCTCCGGACAAAACTGAA<br>R-TCTGCCAGTACCTCCTTGCT	92 %	82 %	ENSSSCT00000025647.4<br>ENSSSCT00000023544.4	Intron 2-3	118 bp
IRF1	F-TGAAGCTGCAACAGATGAGG<br>R-CTTCCCATCCACGTTTGTCT	108 %	112 %	ENSSSCT00000015600.6<br>ENSSSCT00000098417.1	Intron 7-8/8-9	100 bp
IRF4	F-TGAAAATGGTTGCCAGGTG<br>R-GCTTGGCTCTATGGGGATTC	106 %	107 %	ENSSSCT00045027517.1	Intron 4-5	85 bp
IRF5	F-AACCCCGAGAGAAGAAGCTC<br>R-CAAGAAAGCTCCCCTGAGAA	105 %	110 %	ENSSSCT00045018316.1<br>ENSSSCT00045018356.1	Intron 9-10<br>Intron 8-9	88 bp

				ENSSSCT00045018401.1	Intron 8-9	
IRF7	F-GCTCCCCACACTACACCATC<br>R-TCCAACTTCACCAGGACGA	107 %	108 %	ENSSSCT00000014049.4<br>ENSSSCT00000035232.4	Intron 8-9<br>Intron 9-10	91 bp
IRS1	F-CAGCCTCTGGGCAACAGT<br>R-GGAGGTCCTCTGGCTGCT	113 %	117 %	ENSSSCT00000089345.1	-	100 bp
IRS2	F-ACAGGGGATTGTATCGCAAG<br>R-TGGCATGGTCTATGCACTGT	103 %	99 %	ENSSSCT00000043241.2	-	115 bp
KLB (primer pair A)	F-CCTGGGGTGTCACTGAATCT<br>R-TGAGGATCGCTGTACTGTGG	110 %	106 %	ENSSSCT00000028090.3	Intron 4-5	70 bp
KLB (primer pair B)	F-TTCCTCAACCAGGTTCTTCAA<br>R-AACATCCTGCCATTCAAAGC	110 %	105 %	ENSSSCT00000028090.3	Intron 3-4	99 bp
LEP (primer pair A)	F-TGACACCAAAACCCTCATCA<br>R-GTGACCCTCTGTTTGGAGGA	105 %	-	ENSSSCT00000055031.3	Intron 2-3	87 bp
LEP (primer pair B)	F-TGACACCAAAACCCTCATCA<br>R-CCCTCTGTTTGGAGGAGACA	102 %	-	ENSSSCT00000055031.3	Intron 2-3	83 bp
LEPR	F-GCTCAGAATCCCTTCCTCTGT<br>R-CCCCACTCCTTCCATGAATA	101 %	111 %	ENSSSCT00000028665.4<br>ENSSSCT00000075980.2	Intron 17-18<br>Intron 17-18	106 bp
LBP	F-CCCAAGGTCAATGATAAGTTGG<br>R-ATCTGGAGAACAGGGTCGTG	107 %	112 %	ENSSSCT00000032321.4	Intron 13-14	83 bp
LTF	F-GGAAAAGACTGCCCAGACAA<br>R-ACACTCCGTGTTGTCGTTGA	107 %	107 %	ENSSSCT00000030564.3<br>ENSSSCT00000101357.1<br>ENSSSCT00000089932.1	-	78 bp
MBL2	F-AAAGTGGCTTATCTTCGCTCAG<br>R-TCCATTAAAGGACATCTTTTTACCA	88 %	101 %	ENSSSCT00000102163.1	Intron 5-6	82 bp
MMP2	F-TCGCTGGAGATAAGTTCTGGAG<br>R-GGCGTCTGCAATGAGCTT	105 %	102 %	ENSSSCT00000077292.2	Intron 11-12	80 bp
MMP8	F-GGCTGCCTATGAGGATTCTG<br>R-TGAATGTCATAGCCGCTCAG	107 %	107 %	ENSSSCT00000016347.5	Intron 7-8	84 bp
MMP9	F-ACACACGACATCTTCCAGTACC<br>R-GTCCACCTGATTCACCTCGT	112 %	111 %	ENSSSCT00000049521.3	Intron 12-13	96 bp
MPO	F-TGCTACAATGACTCGGTGGA<br>R- GAAGGTGAAGGGTTGGATGA	102 %	116 %	ENSSSCT00000096878.1<br>ENSSSCT00000019199.5<br>ENSSSCT00000035412.3	-	90 bp
MTOR	F-GGGCGATAGACACCCATCTA<br>R- GCAGTCCCCAAAGTCAATGT	110 %	106 %	ENSSSCT00000052098.3<br>ENSSSCT00000055666.3<br>ENSSSCT00000003786.4	Intron 50-51<br>Intron 50-51<br>Intron 50-51	76 bp

NFKB1	F-CCCTGTGAAGACCACCTCTC<br>R-ATCCCGGAGCTCGTCTATTT	112 %	117 %	ENSSSCT00000038683.3	Intron 23-24	82 bp
NFKBIA	F-TGTCTTTGGGTGCTGATGTC<br>R-AGCCCCACACTTCAACAAGA	114 %	117 %	ENSSSCT00000002184.6	Intron 4-5	
NLRP3	F-GACTTTCCAGGAGTTCTTTGCTG<br>R- CCTGGTTTACAAGGCCAAAG	112 %	116 %	ENSSSCT00000054444.2	-	140 bp
NOD1	F-CAGTGGGGTGAAGGTGCTAT<br>R- TACCTGGCTCCGACATCAGT	108 %	107 %	ENSSSCT00000033324.4	Intron 9-10	99 bp
NOD2	F-AGCTCGTGGAACATGCTCTT<br>R- CGTCGGTCAATTTGTTGTTG	101 %	92 %	ENSSSCT00000081424.1<br>ENSSSCT00000099937.1<br>ENSSSCT00000078479.2<br>ENSSSCT00000041200.3	Intron 5-6<br>Intron 5-6<br>Intron 6-7<br>Intron 5-6/6-7	72 bp
NOS2	F-GCAGCTACTGGGTCAAGGAC<br>R-GCTGTTGGTGAACTTCCACTT	108 %	112 %	ENSSSCT00000019325.5<br>ENSSSCT00000055475.3	Intron 20-21<br>Intron 21-22	200 bp
ORM1 (primer pair A)	F-ACCCCCAGTACAATGAGTCG<br>R-TTAACAGCAGGTCAGCAACG	112 %	117 %	ENSSSCT00045028901.1<br>ENSSSCT00045028858.1	Intron 2-3<br>Intron 2-3	210 bp
ORM1 (primer pair B)	F-AGTCCTGAGCCTCCTTCCTC<br>R-GCCGAGCCGATATAATACCA	110 %	107 %	ENSSSCT00045028901.1<br>ENSSSCT00045028858.1	Intron 1-2<br>Intron 1-2	123 bp
PPARG	F-TGCTGTGGGGATGTCTCATA<br>R-CTGCCAACAGCTTCTCCTTC	104 %	103 %	ENSSSCT00000097211.1<br>ENSSSCT00000012672.5<br>ENSSSCT00000037011.3	Intron 4-5<br>Intron 4-5<br>Intron 4-5	74 bp
PPIA	F-CAAGACTGAGTGGTTGGATGG<br>R-TGTCCACAGTCAGCAATGGT	110 %	113 %	ENSSSCT00000018219.5<br>ENSSSCT00000082244.2<br>ENSSSCT00000085168.2	Intron 4-5<br>Intron 5-6<br>Intron 4-5	138 bp
RARRES2	F-TGGAAGAAAGCTGAGTGCAA<br>R-CCTCAGAGTTCAGCTTGATGC	112 %	105 %	ENSSSCT00000057915.1<br>ENSSSCT00000039235.1<br>ENSSSCT00000059850.2	Intron 3-4<br>Intron 4-5<br>Intron 3-4	79 bp
RELA	F-GTGCAGAAAGAGGACATCGAG<br>R-CGTCGGCTTGTGAAAAGG	101 %	103 %	ENSSSCT00000029216.4<br>ENSSSCT00000044525.2<br>ENSSSCT00000014191.6	Intron 7-8<br>Intron 7-8<br>Intron 6-7	76 bp
RPL13A	F-ATTGTGGCCAAGCAGGTACT<br>R-AATTGCCAGAAATGTTGATGC	114 %	110 %	ENSSSCT00000093057.1	Intron 2-3	76 bp
S100A12	F-TGAAGCAGCTGATCACCAAG<br>R-GTCTTGATTGGCATCCAGGT	105 %	103 %	ENSSSCT00000066217.3	Intron 2-3	101 bp

S100A8	F-ATGCTGACGGATCTGGAGAG<br>R-GGCGTGGTAATTCCCTTTCT	105 %	108 %	ENSSSCT00000036517.4	-	84 bp
SCD	F-GGCATTCCAGAATGACGTTT<br>R-GTGGGGATCAGCATCTGTTT	114 %	105 %	ENSSSCT00000074035.2	Intron 3-4	82 bp
SERPINE1	F-CCTGCAAAAGGTGAAGATCG<br>R-ATCACTTGGCCCATGAAAAG	111 %	116 %	ENSSSCT00000026956.4	Intron 8-9/9-10	171 bp
SPP1	TGCTAAAGCCTGACCCATCT	103 %	102 %	ENSSSCT00000010091.5	Intron 4-5	82 bp
SAA2	TGGAGAGCCTACTCGGACAT	111 %	106 %	ENSSSCT00000014601.6<br>ENSSSCT00000102601.1		90 bp
STAT3	F-GCACCTTCCTGCTGAGATTC<br>R-CTGGGTCTTACCGCTGATGT	106 %	110 %	ENSSSCT00000060272.3<br>ENSSSCT00000103644.1<br>ENSSSCT00000096180.1<br>ENSSSCT00000092964.1<br>ENSSSCT00000101406.1<br>ENSSSCT00000057478.3<br>ENSSSCT00000018944.5	Intron 21-22<br>Intron 21-22<br>Intron 22-23<br>Intron 19-20<br>Intron 20-21<br>Intron 21-22<br>Intron 20-21	99 bp
SAA3	F-CAGAGATGGGCATCATTCCT<br>R-TGGCATCGCTGATCACTTTA	110 %	109 %	ENSSSCT00000042621.3<br>ENSSSCT00000084583.2	Intron 2-3/ 3-4<br>Intron 1-2/ 2-3	184 bp
TBP	F-ACGTTCGGTTTAGGTTGCAG<br>R-CAGGAACGCTCTGGAGTTCT	114 %	114 %	ENSSSCT00110006905.1	-	96 bp
TF	F-TAAACAGCAGGCTCAATTTGG<br>R-ATTGGGTGTCATCCCTGAAG	107 %	117 %	ENSSSCT00000071943.2<br>ENSSSCT00000012740.5	Intron 16-17<br>Intron 17-18	104 bp
TGFB1	F-GCAAGGTCCTGGCTCTGTA<br>R-TAGTACACGATGGGCAGTGG	116 %	118 %	ENSSSCT00000036469.4	Intron 6-7	97
TGFB2	F-GACCCCACATCTCCTGCTAA<br>R-ATAGGCTGCATCCAAAGCAC	111 %	99 %	ENSSSCT00000065533.3<br>ENSSSCT00000056615.3	-	94 bp
TLR4	F-TGGTGTCCCAGCACTTCATA<br>R-CAACTTCTGCAGGACGATGA	117 %	106 %	ENSSSCT00000006051.5	-	116 bp
TNF	F-CCCCCAGAAGGAAGAGTTTC<br>R-CGGGCTTATCTGAGGTTTGA	120 %	107 %	ENSSSCT00000104535.2	Intron 1-2/2-3	92 bp
TTR	F-TTGCCTTGGGGAAAACCA<br>R-TGGTGTCCAATTCCACTTTG	98 %	107 %	ENSSSCT00000082485.1	Intron 2-3	96 bp
VCAM	F-CTTGACGTGAAAGGAAGAGAAAG<br>R-GGATGCACAATAGAGCACGA	101 %	99 %	ENSSSCT00000007515.6	Intron 7-8	72 bp
YWHAZ	F-GCTGCTGGTGATGATAAGAAGG	104 %	109 %	ENSSSCT00000006651.4	Intron 3-4	124 bp

	R-AGTTAAGGGCCAGACCCAAT			ENSSSCT00000106709.1<br>ENSSSCT00000037127.3<br>ENSSSCT00000046817.3<br>ENSSSCT00000045155.2<br>ENSSSCT00000099418.1	Intron 5-6<br>Intron 5-6<br>Intron 5-6<br>Intron 2-3<br>Intron 3-4	
